# A cell surface transporter mediates phenanthridine resistance in African trypanosomes

**DOI:** 10.1038/s44259-026-00228-1

**Published:** 2026-07-04

**Authors:** Pieter C. Steketee, Marzuq A. Ungogo, Edith Paxton, Ella Maria Rogerson, Furaha Mramba, Michael C. Pearce, Harriet K. Auty, Jan Van Den Abbeele, Harry P. de Koning, Catarina Gadelha, Michael P. Barrett, Liam J. Morrison

**Affiliations:** 1 The Roslin Institute, Royal (Dick) School of Veterinary Studies, University of Edinburgh, Edinburgh, UK; 2 School of Life Sciences, University of Nottingham, Nottingham, UK; 3 Tanzania Veterinary Laboratory Agency, Dar es Salaam, Tanzania; 4 Global Alliance for Livestock Veterinary Medicines, Edinburgh, UK; 5 School of Biodiversity, One Health and Veterinary Medicine, College of Medical, Veterinary and Life Sciences, University of Glasgow, Glasgow, UK; 6 Trypanosoma Unit, Department of Biomedical Sciences, Institute of Tropical Medicine Antwerp, Antwerp, Belgium; 7 School of Infection and Immunity, University of Glasgow, Glasgow, UK

**Keywords:** Diseases, Microbiology

## Abstract

African animal trypanosomosis poses a significant threat to livestock health and agricultural productivity across sub-Saharan Africa. Isometamidium chloride is the only available drug that is both prophylactic and curative. Despite sustained reports of resistance since the 1970s, a definitive molecular mechanism of resistance remains unresolved in the clinically relevant pathogen species *Trypanosoma congolense*. In this study, the role of a putative drug/metabolite transporter protein, TcoDMT, was validated via the analysis of in vitro-derived mutants, showing that expression levels of this protein correlated strongly with isometamidium sensitivity. Functional analyses revealed that the protein is a cell surface phenanthridine transporter and, notably, copy number variation correlates with isometamidium sensitivity in *T. congolense* field isolates. This study validates, for the first time, a plasma membrane transporter with a defined role in phenanthridine action and resistance, advancing our understanding of drug resistance mechanisms in parasitic protists, and informing strategies to combat animal trypanosomosis.

## Introduction

African trypanosomes are extracellular protozoan parasites that cause both human and livestock disease. Human African Trypanosomiasis (HAT) is prevalent across sub-Saharan Africa, with case numbers decreasing to <1000 annually in recent years, and the disease is currently targeted for elimination of transmission by the WHO^1^. In stark contrast, African Animal Trypanosomosis (AAT) remains a major hindrance to the development of sustainable agriculture in the 37 sub-Saharan African countries where the disease is endemic^2^. Although affecting multiple livestock species, the burden of AAT primarily falls on cattle, and is estimated to result in more than three million cattle deaths annually, with ~90 million at risk, leading to yearly losses of ~$4.5 billion^3^. The three main species of clinical relevance for AAT are *Trypanosoma congolense*, *T. vivax* and to a lesser extent, *T. brucei*. The three species exhibit significant divergence in their biology, including at the genome^4^, transcriptome^5^, proteome^6^ and metabolome^7^ level, as well as in infection phenotypes^8^. Symptoms of infection by the three species are largely indistinguishable, and mixed infections in livestock, in particular cattle and small ruminants such as goats and sheep, and wildlife are common^9^.

There are no vaccines to prevent AAT, although a vaccine target was recently identified for *T. vivax*^10^. Tsetse control is hindered by lack of sustainable infrastructure, leaving chemotherapy as the dominant form of AAT control. Three trypanocides are used in sub-Saharan Africa: the diamidine diminazene aceturate (DA) and the phenanthridines isometamidium chloride (ISM) and homidium bromide (ethidium bromide; EtB)^11,12^. ISM and DA are in widespread use, and although EtB is no longer recommended, it is still sold and used in some areas^13^. ISM is currently the only drug used for both prophylaxis and curative treatment of AAT; prophylaxis being enabled by the long half-life of ISM in the host^9^. ISM is derived from the coupling of EtB with amidinobenzenediazonium chloride, the latter derived from DA, and is marketed as a mixture of four compounds^14^, all of which are bioactive - likely through a shared mechanism^15^.

ISM mode of action studies have predominantly focused on the laboratory-adapted *T. brucei* where it has been hypothesised that ISM interacts with the kinetoplast DNA (kDNA; trypanosome mitochondrial DNA), with strong affinity for double stranded DNA in particular^16,17^. This interaction results in linearization of the kDNA minicircles, with inhibition of a kinetoplast-specific topoisomerase II enzyme proposed as a possible mechanism of action^18^. Unlike ISM, its parental phenanthridine compound (EtB) distributes throughout the trypanosome cell^16^, although it similarly kills trypanosomes by specific interactions with kDNA, in addition to the inhibition of nuclear DNA replication^19^.

The mechanisms governing ISM transport into the cytoplasm and mitochondrion remain unresolved. It is hypothesised that transport into the cytoplasm occurs via facilitated diffusion owing to its positive charge^20^, and reduced cytosolic ISM accumulation, as well as increased efflux have been posited as mechanisms of resistance in *T. brucei*^21^. A possible role in ISM uptake has been proposed for the aminopurine TbAT1/P2 transporter, and although ISM-resistant field isolates of *T. brucei* exhibited mutations in the TbAT1 gene^22^, in vitro uptake studies suggest that the role of TbAT1/P2 is minor^23^. ISM entry in the mitochondrion is thought to rely on the mitochondrial membrane potential (ΔΨ_m_), established by the trypanosome F_0_F_1_-ATPase^20,21^, which functions in reverse to the mammalian ATPase^24^. Agents that depolarise ΔΨ_m_ have been shown to reduce ISM accumulation into the mitochondrion^20,21^. Loss of the kinetoplast results in substantial ISM resistance in *T. brucei*, presumably due to loss of the primary ISM target^21^. However, ISM retains marginal activity against parasites lacking a kinetoplast (dyskinetoplastic), albeit with a significantly increased dose, suggesting the presence of secondary cellular targets^25^. A genome-scale RNAi study in *T. brucei* demonstrated that disruption of vacuolar ATPase (vATPase) was also capable of generating ISM resistance, reflecting the connection between vATPase and mitochondrial F_0_F_1_-ATPase^26^.

There are limited data on ISM modes of action and resistance in *T. congolense*, but it can be predicted that mechanisms are likely to differ from *T. brucei* based on metabolic divergence. An increased reliance upon diverse mitochondrial activity (e.g. substrate-level phosphorylation and an expanded electron transport chain) likely explains why dyskinetoplasty has not been reported in *T. congolense*. However, similar to *T. brucei*, ΔΨ_m_ correlates closely with ISM sensitivity in *T. congolense* genome-reference strain IL3000, which exhibits both a reduced ΔΨ_m_ and increased ISM resistance, compared with other strains^20^. *T. congolense* lacks a TbAT1 orthologue^27^ and to date no ISM transporter has been experimentally validated for either cytoplasmic or mitochondrial uptake.

Earlier studies using field isolates showed that ISM resistance correlated with mutations in a number of *T. congolense* genes, including the topoisomerase II enzyme and an ATP-binding cassette (ABC) transporter^28^. However, correlation with resistance was inconsistent, and a definitive mechanism of ISM resistance has remained elusive^29,30^. A recent study of in vivo generated ISM-resistant *T. congolense* revealed putative determinants via copy number variation (CNV), including ABC transporters, topoisomerase II and a drug/metabolite transporter (TcoDMT)^31^. Expression analysis demonstrated that TcoDMT was downregulated in resistant cells, consistent with a potential role in ISM resistance^31^. Furthermore, one study has reported *T. congolense* isolates exhibiting DMT protein sequence variation, although the relationship of the identified polymorphisms to resistance has not been demonstrated^32^. However, in the absence of functional analyses, the extent to which any of these genetic determinants contribute to ISM resistance has remained unknown. In this study, the role of TcoDMT in ISM-resistance in *T. congolense* and *T. brucei* was functionally evaluated.

## Results

### TcoDMT expression is directly correlated to ISM sensitivity

The recent development of a genetic toolkit for *T. congolense* has enabled manipulation of genes and their functional characterisation, including potential roles in drug mode of action or resistance^33–35^. These advances were exploited to genetically manipulate TcoDMT (gene ID: TcIL3000.A.H_000575200), a single-copy gene in the genome of the reference strain IL3000, which, although exhibiting notable resistance to ISM compared with other strains used in vivo, is the only strain currently amenable to culture. Over-expressor (TcoDMT^OE^) and double knock-out (TcoDMT^dKO^) cell lines were generated, the latter achieved via sequential allelic replacement to also produce a TcoDMT single allele knock-out (hemizygous) intermediate (TcoDMT^sKO^).

**Fig. 1:**
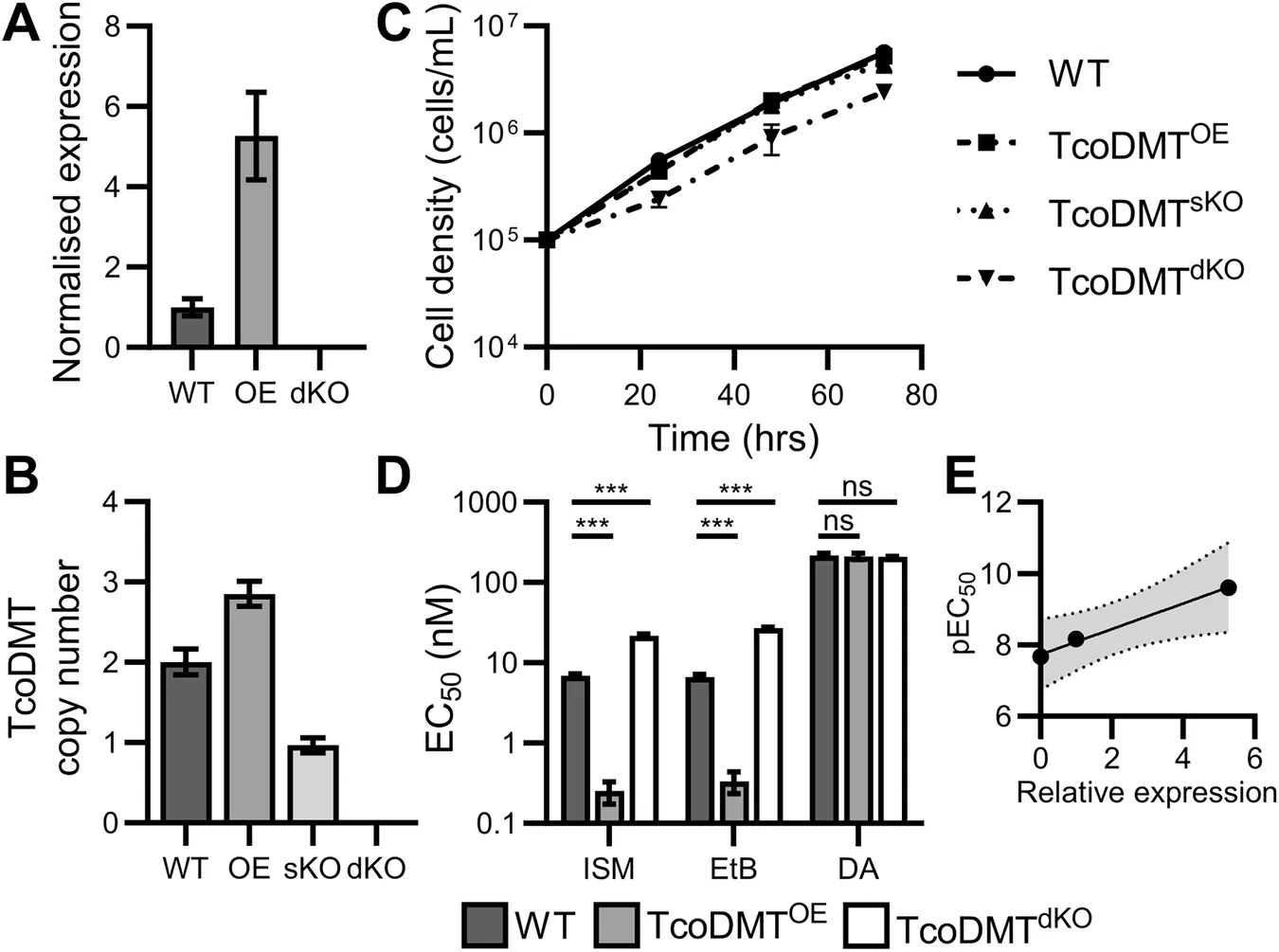
Growth, DMT expression and drug sensitivity in *T. congolense* IL3000 and DMT mutants. **A** qRT-PCR shows 5-fold increased DMT RNA level in TcoDMT^OE^ and loss of signal in TcoDMT^dKO^. Transcript level was normalised to TcoTERT mRNA in each sample. Data represent average of biological triplicate determination, each carried out in technical triplicates (error bars as SEM). **B** Copy number analysis of TcoDMT in WT, TcoDMT^OE^, TcoDMT^sKO^ and TcoDMT^dKO^ mutants. Copy number was analysed via qPCR of genomic DNA and normalised using TcoTERT as endogenous control via the ΔΔCt method. **C** Growth curves of IL3000 wild type (WT), TcoDMT^OE^ and TcoDMT^dKO^ reveal growth defect in the DMT^dKO^. Cells were seeded at 1 × 10^5^ cells/mL and counted by haemocytometer daily for 3 days (error bars indicate SD). **D** Alamar blue drug sensitivity assay reveals variable sensitivity to ISM and EtB, but not DA in WT, TcoDMT^OE^ and TcoDMT^dKO^ cells. Data represent average EC_50_ of biological triplicate measurement determinations, each carried out in technical duplicates (error bars as SEM). Individual statistics performed by unpaired *t*-test, ***P < 0.001; ns not significant. **E** Plot showing the correlation between pEC_50_ (negative logarithm of the EC_50_) and relative expression values calculated in (**A**, **D**). Grey shaded area indicates 95% confidence interval.

TcoDMT expression was elevated in TcoDMT^OE^cells (5.27 ± 1.09-fold vs control) and completely ablated in TcoDMT^dKO^ cells (Fig. 1A), whilst qPCR analysis of genomic DNA confirmed expected gene copy number in the four cell lines tested (Fig. 1B). TcoDMT overexpression did not affect cell growth (doubling time, DT: 12.5 h vs 12.3 h in WT cells; Fig. 1C), but a defect resulting in significantly increased doubling time (DT: 15.6 h vs 12.3 h in WT cells; *P* = 1.20 × 10^−5^, *t*-test) was observed in TcoDMT^dKO^ (Fig. 1C), independent of the presence of selective antibiotics (Fig. S1A). Therefore, ablation of TcoDMT expression in *T. congolense* leads to a fitness cost in vitro.

Drug sensitivity analyses revealed a 27-fold reduction in ISM EC_50_ in TcoDMT^OE^ (*P* = 1.25 × 10^−8^, *t*-test; Table 1 and Fig. 1D). In contrast, a 3.14-fold increase in EC_50_ was observed in TcoDMT^dKO^ compared with WT cells (*P* = 4.12 × 10^−9^; Table 1 and Fig. 1D), indicating increased ISM resistance in dKO mutants. Thus, in vitro, TcoDMT expression levels correlate linearly with ISM sensitivity in *T. congolense* (r^2^ = 0.99; Fig. 1E). Minor, but not significant, changes in ISM resistance were observed in TcoDMT^sKO^, and this line was therefore not included in downstream analyses (Fig. S1B). Knock-down of TcoDMT was also attempted using a *T. congolense* IL3000 single-marker cell line (TcoSM) that constitutively expresses T7 RNA polymerase and Tet Repressor^33^. However, RNAi penetrance is low in *T. congolense*^7,33^, and only 20–40% reduction in TcoDMT mRNA levels was observed, which did not affect ISM sensitivity (Fig. S2). The RNAi and TcoDMT^sKO^ data jointly suggest that near complete ablation of TcoDMT expression was required for increased ISM resistance in the in vitro *T. congolense* strain IL3000.

**Table 1:** DMT mutant EC_50_s for ISM, DA and EtB

Species	Cell line	ISM			DA			EtB		
		EC_50_ (nM)	FC vs ctrl	P-val vs ctrl	EC_50_ (nM)	FC vs ctrl	P-val vs ctrl	EC_50_ (nM)	FC vs ctrl	P-val vs ctrl
*T. congolense*	WT	6.88 ± 0.39	-	-	216.4 ± 14.5	-	-	6.7 ± 0.5	-	-
*T. congolense*	TcoDMT^OE^	0.25 ± 0.08	0.04	1.25 × 10^−8^	212.4 ± 18.5	0.98	>0.05	0.3 ± 0.1	0.04	2.07 × 10^−7^
*T. congolense*	TcoDMT^dKO^	21.59 ± 1.14	3.14	4.12 × 10^−9^	208.1 ± 4.1	0.96	>0.05	26.9 ± 0.8	4.01	2.25 × 10^−11^
*T. brucei*	WT	141.78 ± 26.69	-	-	80.77 ± 8.56	-	-	1,343.3 ± 43.8	-	-
*T. brucei*	TbDMT^OE^	9.21 ± 2.6	0.10	2.57 × 10^−4^	79.42 ± 16.39	0.85	>0.05	760.7 ± 23.0	0.57	2.27 × 10^−5^
*T. brucei*	Tb-TcoDMT^OE^	9.20 ± 0.21	0.06	0.02	79.40 ± 16.40	1.02	>0.05	Not tested	-	-
*T. brucei*	TbDMT RNAi (uninduced)	289.77 ± 5.71	-	-	68.50 ± 1.89	-		859 ± 23.9	-	-
*T. brucei*	TbDMT RNAi (induced)	337.25 ± 18.06	1.16	0.03	54.27 ± 2.18	0.79	0.001	761.6 ± 15.7	0.89	0.007
*T. brucei*	TbDMT Cas-9 dKO (uninduced)	573.10 ± 66.32	-		149.55 ± 13.21	-		Not tested	-	-
*T. brucei*	TbDMT Cas-9 dKO (induced)	650.04 ± 43.99	1.13	>0.05	167.48 ± 21.78	1.12	>0.05	Not tested	-	-

A summary of drug sensitivity data for the *T. congolense* and *T. brucei* DMT mutants described in this study. Fold changes and P-values are in relation to WT parental cells, or in the case of *T. brucei* RNAi and Cas9, in relation to uninduced controls. *Ctrl* control, *FC* fold change, *P-val* P-value.

Given that ISM consists of an EtB base coupled to the *m*-amidinobenzenediazonium chloride moiety derived from DA^36^, sensitivities for all *T. congolense* cell lines to EtB and DA were determined in parallel (Fig. 1D). Whilst sensitivity or resistance to EtB was altered to approximately the same extent in the TcoDMT mutants, when compared with changes in ISM sensitivity (*P* = 2.01 × 10^−7^ and 0.03 for WT vs TcoDMT^OE^ and WT vs DMT^dKO^, respectively; Table 1 and Fig. 1D), DA sensitivity remained unchanged, irrespective of changes in TcoDMT expression (Table 1 and Fig. 1D). Thus, the association of TcoDMT with ISM sensitivity is due to interactions with the EtB moiety of the ISM molecule, and TcoDMT does not confer sensitivity/resistance to DA.

### TcoDMT is a cell surface protein

**Fig. 2:**
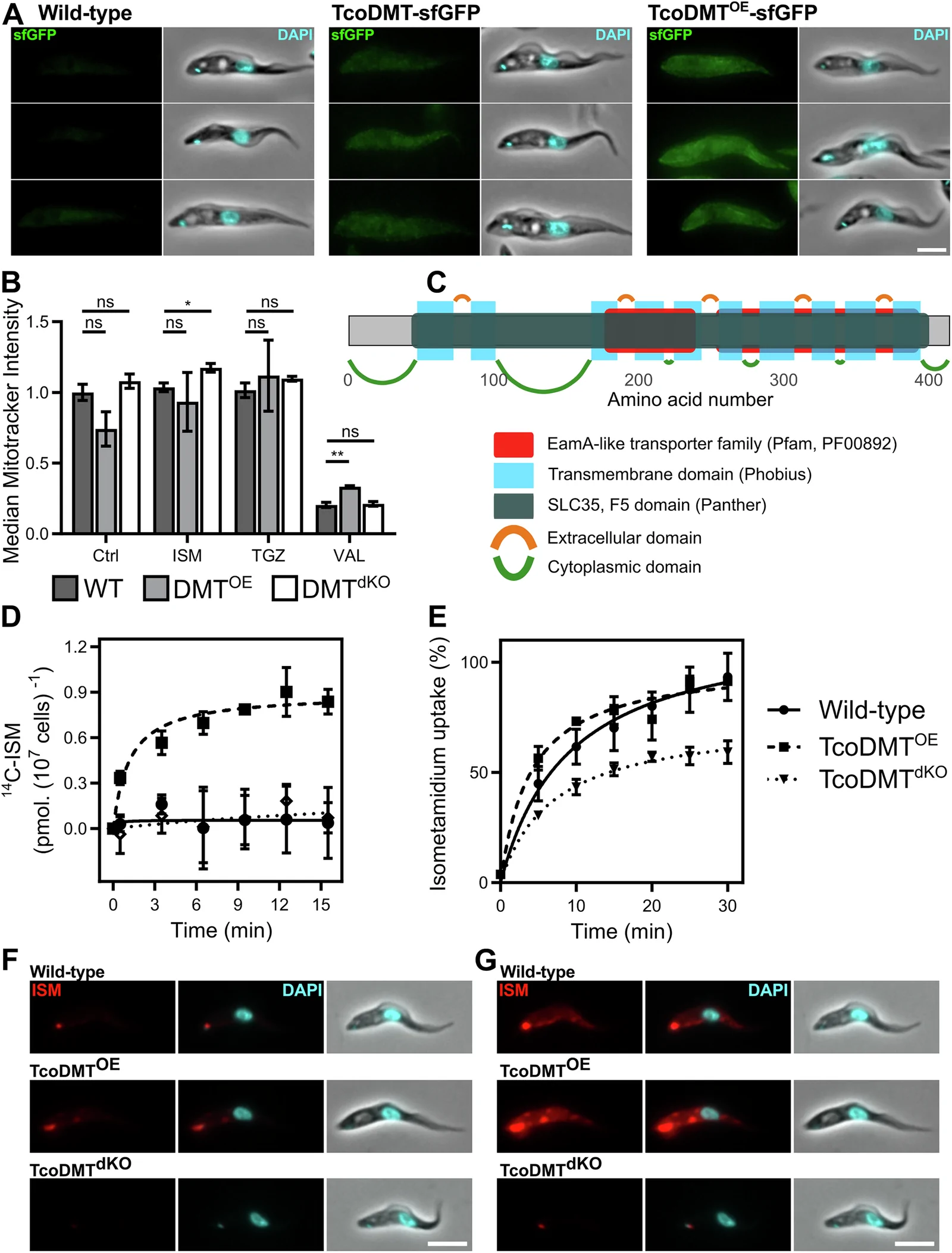
TcoDMT is a cell surface ISM transporter that does not impact ΔΨ_m_. **A** TcoDMT was N-terminally tagged with superfolder (sf)GFP^94^ at the endogenous locus and ectopically at the tubulin locus. For both lines, native fluorescence (green, left panel) was observed on the cell periphery, indicative of parasite cell surface. DAPI (cyan) was used to visualise the nuclear and mitochondrial DNA. Three examples are shown for wild-type cells (left), TcoDMT^sfGFP^ (middle) and TcoDMT^OE-sfGFP^ (right). Scale bar represents 5 µm. **B** Mitotracker fluorescence intensity in WT, TcoDMT^OE^ and TcoDMT^dKO^, indicative of mitochondrial uptake of Mitotracker that correlates with ΔΨ_m_. Cells were left untreated or treated with 500 nM ISM for 3 h. Cells treated with 20 µM troglitazone or 200 nM valinomycin for 1 h were included as positive and negative control, respectively. All samples were incubated with 0.05 µM Mitotracker and fixed prior to detection of fluorescence intensity. The mean of 3 biological replicates was normalised to the untreated WT control (error bars represent SEM). *P < 0.05; **P < 0.005; ns, not significant by Unpaired Student’s *t* test compared to the untreated WT control. **C** Domain analysis of the TcoDMT protein. The protein sequence of TcoDMT was analysed via InterProScan^84^, highlighting 10 predicted transmembrane domains (light-blue), as well as two predicted EamA-like transporter domains (PF00892; red) and an SCL35, F5 domain as predicted by Panther (dark grey). Also predicted were numerous cytoplasmic (orange) and inner mitochondrial (green) domains. **D** Uptake assays with 1 µM ^14^C-ISM in IL3000 WT and DMT mutants. Data represent average of biological triplicate determinations, each carried out in technical triplicates (error bars as SEM). **E** Fluorescence-based uptake of 10 µM ISM in IL3000 WT and DMT mutants. Data represent average of two biological repeats each carried out in technical triplicates (error bars as SEM). **F** Immunofluorescence microscopy analysis of ISM uptake into *T. congolense* IL3000 WT and TcoDMT mutant cells. WT, TcoDMT^dKO^ and TcoDMT^OE^ cells were incubated with 10 µM ISM for 30 min prior to slide preparation for imaging. For WT and TcoDMT^OE^, ISM localised to the kinetoplast, with staining also observed in the mitochondrion in the latter. Under these brightness settings, no ISM was detected in TcoDMT^dKO^ cells. **G** The same images as in ‘B’, with increased brightness in the ISM channel to highlight that even in TcoDMT^dKO^ cells, ISM accumulates in the kinetoplast exclusively, albeit at much lower abundance. Scale bar represents 5 µm.

To assess the cellular location of TcoDMT, the gene was endogenously-locus tagged at its N-terminus using codon-optimised superfolder GFP (sfGFP)^37^, and the resulting cell line analysed by fluorescence microscopy. The sfGFP signal was relatively weak, albeit with detectable accumulation at the cell surface (Fig. 2A, S3A). Analysis of pixel intensity supported the hypothesis that TcoDMT is a cell surface protein (Fig S3B). To increase TcoDMT expression levels for improved visualisation, an N-terminally tagged ectopic copy was inserted into the tubulin locus (analogous to the TcoDMT^OE^ line). The TcoDMT^OE-sfGP^ line exhibited clear accumulation of sfGFP at the cell surface (Fig. 2A, S3A), thus confirming that TcoDMT is a plasma membrane protein.

Previous studies have reported a correlation between mitochondrial membrane potential (ΔΨ_m_) and ISM sensitivity in *T. brucei*^21^, and a putative link in *T. congolense*^20^. Whilst a cell surface transporter is unlikely to impact upon ΔΨ_m_, we nevertheless assessed ΔΨ_m_ in the TcoDMT mutants (Fig. 2B). Untreated TcoDMT^OE^ exhibited a slight, but not significant (*P* > 0.05) reduction in ΔΨ_m_ (0.74 ± 0.1 arbitrary units [AU], compared with 1.00 ± 0.1 AU in untreated WT cells; Fig. 2B). While ISM treatment of *T. brucei* results in a rapid ΔΨ_m_ reduction^21^, no effect was observed after a two-hour ISM treatment of *T. congolense* WT or mutant cells (Fig. 2B). Notably, *T. congolense* IL3000 exhibits a lower ΔΨ_m_ compared to ISM-sensitive *T. congolense* strains^20^. Nevertheless, treating all cell lines with valinomycin, a mitochondrial membrane depolariser, resulted in a > 50% reduction in ΔΨ_m_ (Fig. 2B); troglitazone, known to hyperpolarise ΔΨ_m_ in *T. brucei*^38^, had no effect. These data suggest that TcoDMT plays no active role in ΔΨ_m_ maintenance.

TcoDMT is a putative EamA-like transporter encoding 10 transmembrane domains and also harbouring an SLC35F, family 5 (SLC35F5) domain (Fig. 2C). The human orthologue of SLC35F5 is an orphan transporter implicated in anti-cancer chemotherapeutics resistance^39,40^. The predicted *T. brucei* orthologue, TbDMT (Gene ID: Tb927.8.1460), was previously predicted as a nucleotide sugar transporter (TbNST)-7, where TbNST1-4 were experimentally characterised as functional nucleotide sugar transporters, while TbNST7 was not functionally characterised^41^. Phylogenetic analysis confirmed that TcoDMT and TbDMT are closely related to the F5 subgroup of the SLC35 superfamily, and are syntenic orthologues (Fig. S4A). A DMT orthologue was also identified in *T. vivax* (Gene ID: TvY486_0800880) and *Leishmania major* (gene ID: LmjF.07.0400; Fig. S4A). However, no orthologues were identified in *T. cruzi*, nor in the intracellular apicomplexans *Toxoplasma gondii* and *Plasmodium falciparum*.

A ColabFold^42^ TcoDMT model was shown to be most similar to predicted models of SLC35F5 proteins from *T. brucei*, *L. major*, animals and yeast (Fig. S4B–D and Supplementary Data 1). An untargeted metabolomics approach was carried out to determine whether changes in abundance to any metabolite could be identified in TcoDMT^OE^ and TcoDMT^dKO^ when compared to WT cells (Fig. S5). However, despite several differences appearing in metabolites between WT and TcoDMT^OE^ lines, no clear cut substrate specificity could be attributed using this approach, leaving the normal physiological role of TcoDMT unresolved at this time (Fig. S5 and Supplementary Data 2).

### TcoDMT is an ISM transporter

To date, no ISM transporters have been identified in African trypanosomes. To determine whether TcoDMT could transport ISM, uptake assays were carried out using radioactive (^14^C)-ISM (Fig. 2D), as well as taking advantage of the intrinsic fluorescence of ISM (which is dependent on kDNA binding) to analyse mitochondrial uptake (Fig. 2E). No statistical difference was found in the uptake of 1 mM ^14^C-ISM between TcoDMT^dKO^ and WT cells, where uptake was minor and remained flat over 15.5 min (Fig. 2D). However, the uptake of ^14^C-ISM in DMT^OE^ was rapid (significantly increased in 30 s; *P* < 0.05 for all time points) and plateaued within 3.5 min, indicating a rapid equilibration between the extracellular and cytosolic ISM concentrations (Fig. 2D). At 16 min, TcoDMT^OE^ accumulated approximately 22-fold higher ISM than WT control. These findings were validated using measured fluorescence-based uptake, which depends on the binding of the drug to the trypanosome kinetoplast^17,21^ (Fig. 2E). A reduction in ISM uptake was observed in DMT^dKO^, which reached 59.3 ± 5.1% at 30 min compared with 93.4 ± 10.8% in WT cells (Fig. 2E). Fluorescence-based uptake reached a plateau, indicative of an equilibrative transporter mechanism, after 20 min in all cell lines (Fig. 2E), and although the equilibrium was reached sooner in TcoDMT^OE^ compared to WT, the difference was not statistically significant (*P* > 0.05) at any single time point. Fluorescence microscopy showed that the ISM-derived fluorescent signal was dependent on uptake into the kinetoplast in all samples, including TcoDMT^dKO^ cells (Fig. 2F, E).

### DMT plays a role in ISM and EtB sensitivity in *T. congolense*, but not *T. brucei*

**Fig. 3:**
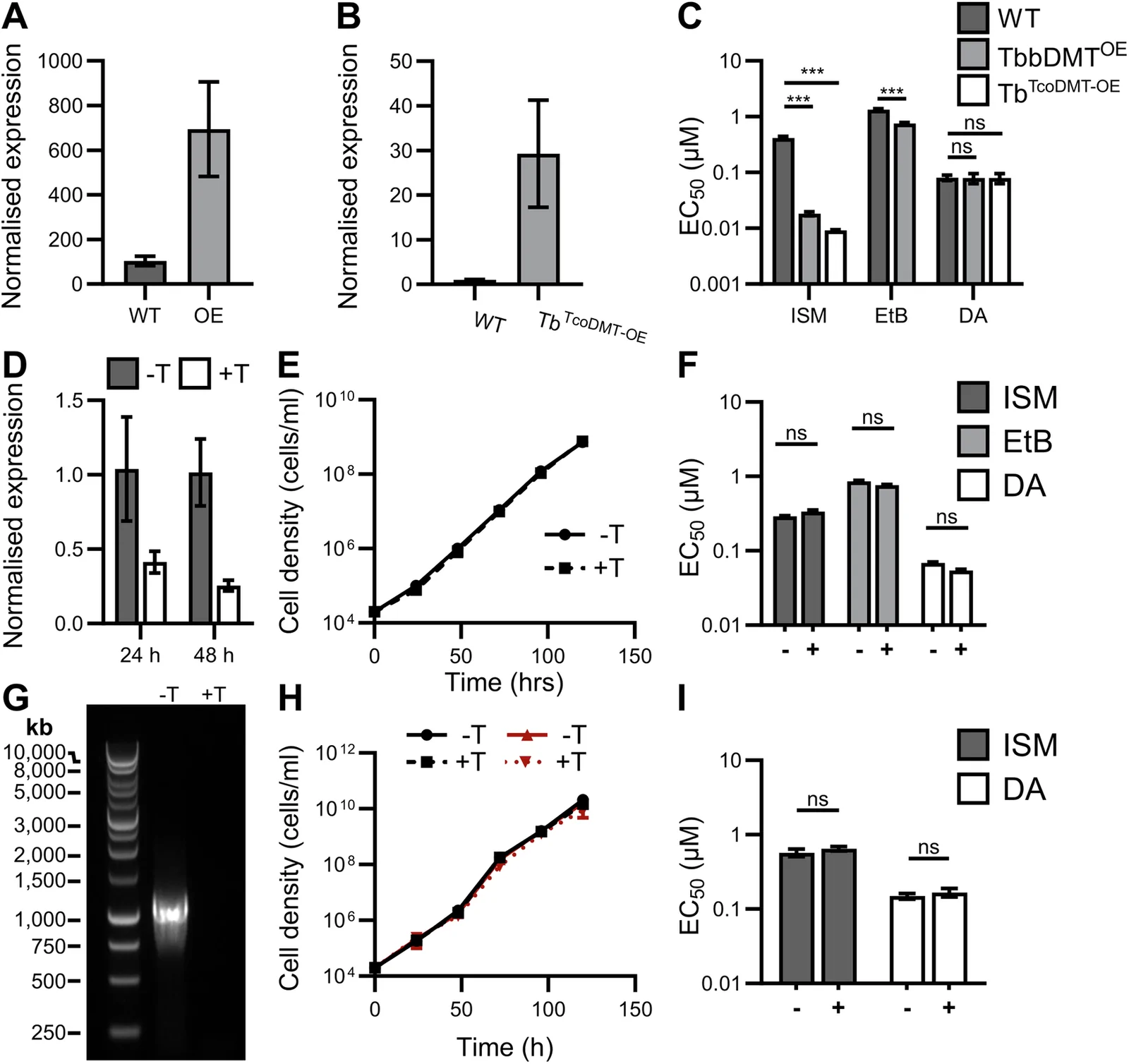
Analysis of the impact of TbDMT expression on ISM sensitivity. **A** qPCR analysis of TbDMT gene expression in WT and TbDMT^OE^ *T. brucei* cell lines shows approximately seven-fold increased expression in TbDMT^OE^. **B** qRT-PCR confirms transgenic expression of the TcoDMT gene in *T. brucei* cells (Tbb^TcoDMT-OE^). **C** Drug sensitivity analysis reveals increased sensitivity to ISM (fifteen-fold) and EtB (two-fold), but not to DA in TbDMT^OE^ cells. Additionally, Tbb^TcoDMT-OE^ cells also exhibit increased sensitivity to ISM and EtB, but also not to DA. **D** qPCR analysis of TbDMT gene expression in TbDMT RNAi cells indicates ~60% and ~75% reduction in DMT mRNA at 24 h and 48 h post-induction via the addition of 1 µg/mL tetracycline, respectively. **E** TbDMT RNAi lines display no growth defects in vitro upon induction. **F** Drug sensitivity analysis of TbDMT RNAi cells reveals no significant changes in ISM, EtB or DA sensitivity upon RNAi induction. **G** PCR amplification of the TbDMT gene in TbDMT-Cas9 cells confirms knock-out of the TbDMT ORF (~1.2 kB) following tetracycline induction. **H** Cas9-mediated knock-out of TbDMT was induced via addition of 1 µg/mL tetracycline. Two different sgRNA sets were assessed (set 1: black lines; set 2: red lines). Growth rates of TbDMT-KO cells remained similar to uninduced cells. **I** Cas9-mediated TbDMT knock-out cells exhibit no significant differences in ISM, EtB or DA sensitivity compared to uninduced controls. All qPCR data represents normalised expression relative to WT using TERT as endogenous control, and experiments were carried out with biological triplicates each in technical triplicates (averages shown, error bars as SEM). Cumulative growth rates were determined through daily manual counts of 3 clones (error bars as SEM) seeded at 2 × 10^4^ cells/mL and maintained at the log phase of growth through 1:10 passage. Drug sensitivity data represents average EC_50_ after 72 h exposure to drugs of biological triplicate determination each carried out in technical duplicates (error bars as SEM). *, *P* < 0.05; **, *P* < 0.01***, *P* < 0.005; ns not significant by Unpaired Student’s *t* test compared to WT control. CRISPR-Cas9-mediated DBS was carried out using 2 separate sgRNA sets, both of which showed similar result.

TcoDMT shares 59.7% protein sequence identity with TbDMT, and 50.4% with the *T. vivax* syntenic orthologue (Tv486_0800880; Fig. S6). TbDMT was not identified to be an essential protein in RNAi screens^43^, nor was TbDMT an identified determinant of ISM resistance in a *T. brucei* genome-wide RNAi screen carried out under ISM selection^26^, suggesting that the role of DMT in ISM resistance may be particular to *T. congolense*. To investigate this further, a TbDMT overexpression line (TbDMT^OE^), a *T. brucei* mutant expressing TcoDMT from the tubulin locus (Tb^TcoDMT-OE^), as well as gene knock-down (TbDMT^RNAi^) and knock-out (inducible CRISPR/cas9; TbDMT^dKO^) mutants were generated, in order to assess any role of DMT in ISM-resistance in *T. brucei* (Fig. 3).

Overexpression of TbDMT (Fig. 3A) resulted in a 22-fold increase in ISM sensitivity (*P* = 0.0176; Table 1 and Fig. 3C), with no significant differences in DA sensitivity (*P* = 0.95; Table 1 and Fig. 3C). However, TbDMT^OE^ cells only showed a 1.8-fold increase in sensitivity to EtB (*P* = 0.0003; Table 1 and Fig. 3C). Additionally, RNAi-mediated knockdown of TbDMT (~75% reduction in mRNA, Fig. 3D) did not affect parasite growth (Fig. 3E), nor sensitivity to either DA or ISM (Fig. 3F). To rule out the possible reversal of induced knockdown over the period of drug assay, two independent inducible TbDMT-Cas9 cell lines were generated using separate sgRNAs as previously described^44^. Tetracycline induction achieved detectable double-strand breaks within 5 days (Fig. 3G). However, there was no change in growth rate (Fig. 3H), nor differential sensitivity to DA or ISM between the uninduced cells and TbDMT knock-out mutants (Fig. 3I). TcoDMT function was further validated through transgenic expression in the *T. brucei* tubulin locus, confirmed by qPCR (Fig. 3B). Tb^TcoDMT-OE^ clones showed approximately 45-fold increase in ISM sensitivity (*P* = 0.0154; Table 1, Fig. 3C), but no significant difference in the sensitivity to DA compared to the WT control (Fig. 3C).

### DMT copy number is associated with ISM sensitivity in field isolates

**Fig. 4:**
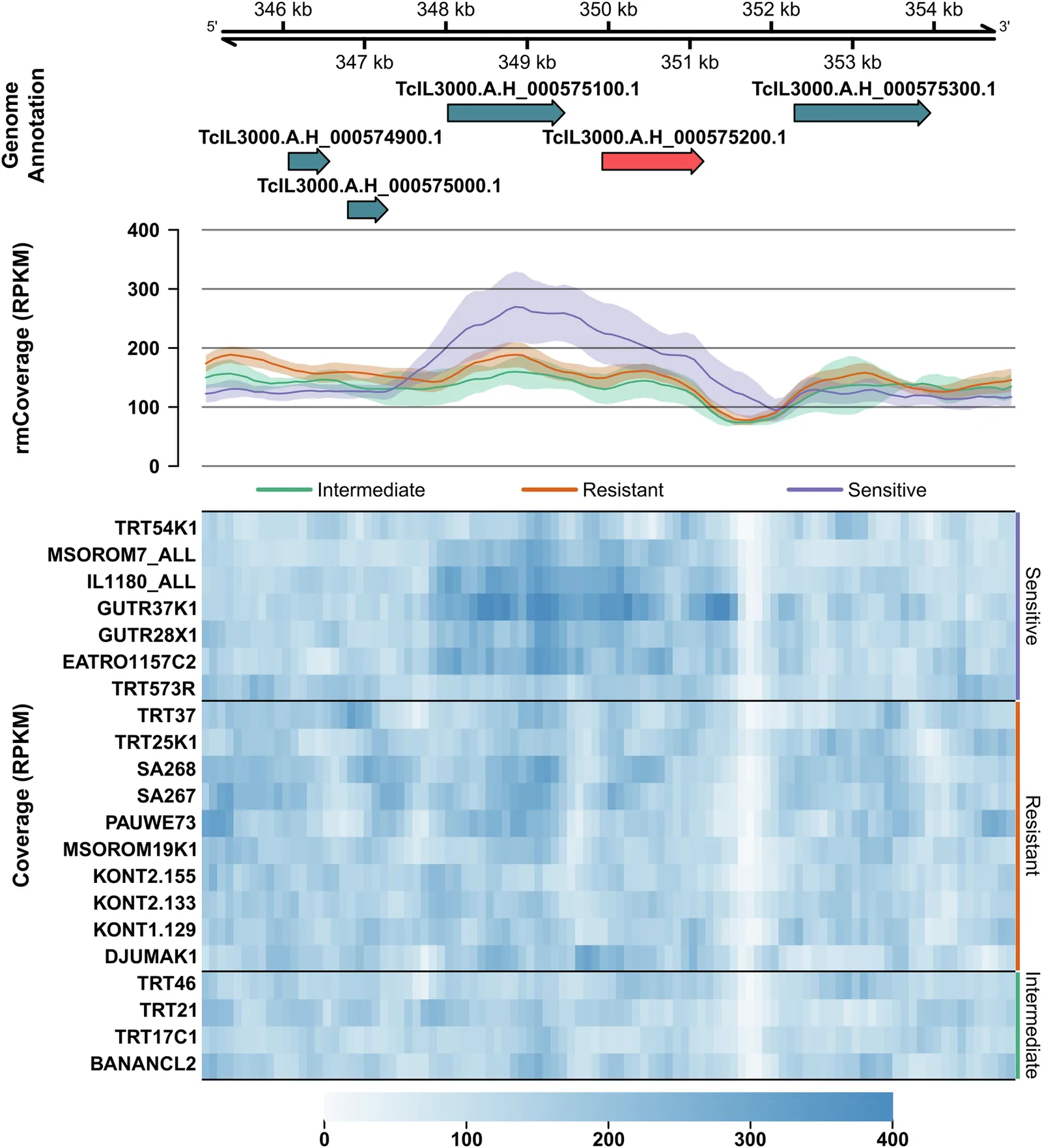
Coverage of *TcoDMT* in *T. congolense* genome. Genomic sequencing data was obtained from previous studies^45^. Of the 56 field isolates sequenced by Tihon and colleagues, 21 were previously characterised in terms of ISM resistance (Supplementary Data 3), and these were taken forward for further analysis. Based on ISM-resistance metadata, samples were divided into three groups based - Sensitive, Intermediate Sensitivity (stemming from two studies^28,95^; Supplementary Data 3) and Resistant - and the rolling mean coverage (rmCoverage; mean coverage in RPKM at 100 bp intervals) was calculated. rmCoverage for the TcoDMT locus was higher in ISM-sensitive isolates. All sequencing data was aligned to the *T. congolense* IL3000 genome (TriTrypDB, version 51) which contains a single DMT gene copy. S, sensitive; R, resistant; I, intermediate sensitivity. Chromosome and genes in proximity of the TcoDMT locus are shown at the top of the figure (red arrow in the Genome Annotation panel indicates TcoDMT). The second panel shows average rmCoverage (with confidence intervals shown in lighter colour) for the three sample groups, whilst the lower panel shows coverage at 100-bp intervals for each field isolate included in this analysis divided into the three groups.

To verify whether DMT copy number is associated with ISM sensitivity in the field, publicly available genome sequences^45^ of *T. congolense* field isolates with documented ISM sensitivity phenotypes were analysed (Fig. 4 and Supplementary Data 3). Resistance was defined if relapse occurred in 80–100% of cases of infected mice treated with 1 mg/kg ISM, where intermediate resistance was defined if relapse occurred in 40–80%, as elaborated in the original studies (references in Supplementary Data 3).

Sequences from 21 isolates were aligned to the *T. congolense* IL3000 genome sequence (which encodes one DMT gene copy) and average reads per 100 bases (rolling means coverage; rmCoverage) were plotted (Fig. 4). Increased read coverage associated with the *TcoDMT* gene (TcIL3000.A.H_000575200), as well as in an upstream hypothetical ORF (TcIL3000.A.H_000575100), was detected in ISM-sensitive isolates. For isolates classed either as intermediate or fully resistant to ISM, rmCoverage was reduced compared to sensitive isolates, indicating that *TcoDMT* copy number positively correlates with ISM sensitivity. These data were compared to several other regions of the genome encompassing well characterised single-copy loci in *T. congolense* IL3000, aldolase (*TcoALD*), fructose-1,6-bisphosphatase (*TcoFBP*) and telomerase reverse transcriptase (*TcoTERT*; Fig S7). Here, no differences in rmCoverage were found between the three *T. congolense* ISM-sensitivity phenotype groups (Fig. S7), suggesting that the observed increased read coverage in the ISM-sensitive isolates was particular to the *TcoDMT* locus.

## Discussion

Drug treatment failure remains a significant threat to the chemotherapeutic control of AAT, impacting upon animal health and welfare as well as the development of sustainable agriculture in sub-Saharan Africa^46–48^. ISM is the most common chemotherapy alongside DA, and is also used as a prophylactic, heightening the likelihood of drug resistance acquisition by the parasite, especially after over six decades of widespread use^11^. The molecular mechanisms of ISM resistance, especially in the clinically relevant parasite species *T. congolense*, remain poorly understood, despite multiple reports of resistance leading to reduced ISM accumulation^20–22,31,49,50^.

Biological understanding of drug resistance mechanisms in *T. congolense* is lacking. So far, the best characterised putative mechanism for ISM resistance in this important pathogen is reduced ISM accumulation due to reduced ΔΨ_m_, limiting ISM import into the mitochondrion^20^ (similar to observations in *T. brucei*). The study of ISM resistance in *T. congolense* is further hindered by a lack of culturable field isolates, as the main lab strain, IL3000, is inherently ISM resistant and already exhibits a reduced ΔΨ_m_, when compared to sensitive strains^20^. However, a recent study bypassed this challenge by generating resistance via iterative drug treatment of an ISM-sensitive strain (KTT/MSOROM7C1^51^) during mouse infections^31^. A key finding in this study was the observed variation in copy number of several genes, including a putative drug/metabolite transporter (TcoDMT), an ABC transporter and the mitochondrial type II topoisomerase^31^. Whilst the type II topoisomerase was historically implicated in ISM resistance, mutations in field isolates were shown to have no impact on ISM sensitivity in subsequent studies^29^, and copy number variation in this protein was unlikely to be related to resistance. In contrast, TcoDMT gene expression was reduced in ISM resistant cells, suggesting TcoDMT protein abundance is likely to play a role in ISM resistance^31^.

In this study, validating the role of TcoDMT in ISM sensitivity was made possible due to recently-developed genetic tools^33,34^ enabling the generation of TcoDMT expression mutants. Whilst TcoDMT overexpression led to a significant increase in ISM and EtB sensitivity, loss of TcoDMT expression led to increased ISM and EtB resistance, thus confirming that TcoDMT is a genetic determinant of ISM and EtB resistance in *T. congolense*. Altered expression of TcoDMT had no impact on sensitivity to DA, suggesting a structure-activity relationship between TcoDMT and the EtB-base moiety of the ISM molecule, as well as supporting previous observations in this species of ISM resistance being associated with high levels of cross-resistance to EtB, but not DA^52^. Notably, field isolates resistant to both ISM and DA have been detected in the field^53^, which are likely to be the result of multiple independent mutations rendering the parasite resistant to both drugs (‘co-resistance’ rather than ‘cross-resistance’)^12^. The small, yet significant increase in resistance in TcoDMT^dKO^ mutants could be explained by the IL3000 strain already being ISM-resistant^20^ and TcoDMT being a single-copy gene. The fact that TcoDMT^dKO^ cells retain low levels of ISM retention could indicate that there are additional uptake routes that, at the minimal level of TcoDMT expression, significantly contribute to the residual activity. Indeed, uptake assays and fluorescence microscopy suggest that, even in the absence of TcoDMT, there is some degree of ISM uptake.

*T. brucei* studies have linked mutations in the F_1_-subunit γ of the F_1_F_0_-ATP synthase to ISM resistance, as these mutations allow bloodstream form parasites to compensate for loss of the kinetoplast with a concurrent reduction in ΔΨ_m_^21,54,55^. In contrast to *T. brucei*, dyskinetoplasty has not been reported for *T. congolense* to the best of our knowledge, and this is consistent with elevated mitochondrial activity in the bloodstream form of this species compared to *T. brucei*, suggesting the kinetoplast is likely to be essential^7,56^. Modifying TcoDMT expression had no effect on ΔΨ_m_, indicating that the ISM resistance mechanism uncovered here operates independently of membrane potential.

Instead, the evidence presented here support a role for TcoDMT in cytosolic import of ISM. The protein localises to the cell surface, and uptake assays demonstrate altered drug accumulation when expression is modified. Fluorescence-based assays exploit ISM binding to the kDNA, thereby directly reporting mitochondrial accumulation. TcoDMT^dKO^ cells displayed reduced mitochondrial signal, consistent with impaired drug accumulation. TcoDMT overexpression did not increase steady-state mitochondrial accumulation over 30 min, although initial rate of influx appeared enhanced. In contrast, whole cell uptake measured using ^14^C-ISM was substantially increased in TcoDMT^OE^ cells, while ablation of TcoDMT expression had no significant effect on ^14^C-ISM accumulation.

**Fig. 5:**
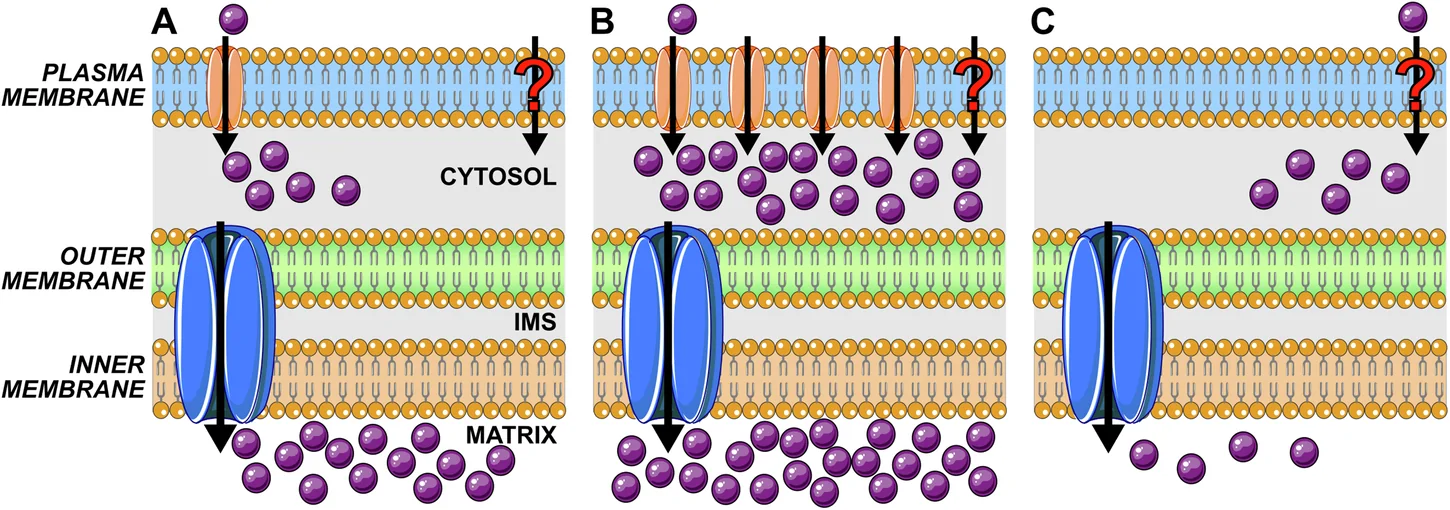
Proposed mechanism of the role of TcoDMT in ISM sensitivity and resistance. **A** WT *T. congolense* IL3000; **B** TcoDMT^OE^; **C** TcoDMT^dKO^. Under normal conditions, there are two concentration gradients for ISM, that between the extracellular environment and the cytosol, and that between the cytosol and mitochondrial matrix. ISM uptake at the cell surface is mediated by TcoDMT, whilst mitochondrial uptake is mediated via mitochondrial membrane potential. This means that both concentration gradients are maintained until the mitochondrion is saturated. When TcoDMT expression is increased (via CNV, or in vitro), ISM is imported into the cytosol at higher rates, with mitochondrial uptake becoming the rate limiting step. The mitochondrion therefore becomes saturated at a faster rate. When TcoDMT expression is abolished, cell surface uptake is interrupted, resulting in a knock-on effect of reduced mitochondrial uptake. Schematic generated in Inkscape using graphics from Bioicons.

The likely explanation for these data is the consideration that ISM must traverse three membranes - the plasma membrane, and the outer and inner mitochondrial membranes - to reach the kDNA. Two concentration gradients therefore exist: (i) that between the extracellular environment and the cytosol, and (ii) that between the cytosol and mitochondrial matrix. The following model is therefore proposed (Fig. 5), whereby TcoDMT mediates ISM import across the plasma membrane, with the plasma membrane potential (ΔΨ_pm_) providing the drive force for the dicationic drug, analogous to the role of ΔΨ_m_ in mitochondrial accumulation.

Under WT conditions (Fig. 5A), ISM uptake proceeds until mitochondrial binding sites are saturated. Increasing TcoDMT (Fig. 5B) enhances the rate of cytosolic import and thereby accelerates mitochondrial equilibration, but does not substantially alter steady-state accumulation, which is ultimately constrained by downstream steps, including kDNA saturation. Conversely, disruption of TcoDMT (Fig. 5C) slows cytosolic entry, reducing the rate of mitochondrial accumulation and resulting in a modest loss of sensitivity.

Importantly, although active ISM concentrations are in the nM-µM range, the uptake assays were performed with either 1 µM ^14^C-ISM or 10 µM ISM by fluorescence, due to detection limits. These experimental conditions will overestimate the contribution of a secondary, low-affinity ISM transporter. At therapeutic concentrations, TcoDMT may contribute proportionally more to uptake if it has a higher affinity than secondary transporters. Similarly, in *T. brucei*, the principal determinant for pentamidine sensitivity is TbAQP2, first characterised as the High Affinity Pentamidine Transporter. Although TbAT1/P2 exhibits a higher pentamidine transport capacity (V_max_) TbAQP2 dominates at therapeutic concentrations owing to its very low K_m_^57^.

Importantly, complete gene loss seems only to be possible in *T. congolense* under in vitro conditions, as all field isolates appear to possess at minimum one TcoDMT copy – presumably because the observed growth defect reduces fitness of null mutants in vivo, such that low copy number mutants which are resistant to the selective pressure of ISM will be preferentially selected under these conditions. Therefore, it is likely that in the field, TcoDMT CNV is the main driver of resistance, rather than complete loss of expression.

Indeed, analysis of genomic data from *T. congolense* field isolates corroborates this hypothesis, suggesting that TcoDMT is likely to play a role in ISM resistance in a field setting. CNV of genes or chromosomal regions has previously been associated with changes in drug sensitivity in trypanosomatids. For example, CNV in nitroreductase (NTR)-1 correlated with nifurtimox resistance in *T. brucei*^58^ and *T. cruzi*^59^, and a recent study characterised CNV in trypanosomal serine carboxypeptidases in the acquisition of benzoxaborole resistance^34^. Whilst ISM resistance is likely to arise via multiple independent (or cumulative) mechanisms^21,26,31^, the correlation of TcoDMT copy number with susceptibility/resistance across multiple field-isolated strains from diverse geographic and temporal origins suggests that TcoDMT CNV is a mechanism that is frequently involved in ISM resistance.

Whilst DMT has a capacity for both ISM and EtB uptake, this study was not able to experimentally identify the physiological substrate of this transporter. DMT is a homologue of SLC35F5, a class of solute carrier generally known as nucleotide sugar transporters, although the SLC35F subfamily are largely regarded as orphan transporters with substrate-specificity yet to be determined in most cases, although *H. sapiens* SLC35F5 was recently proposed to carry choline^60^, and queuine and queuosine (SLF35F2^61^), as well as thiamine (SLC35F3^62^) have all been proposed as substrates recently. Given its localisation on the cell surface, DMT is unlikely to transport nucleotide sugars (trypanosomes synthesise these in the glycosome^63^, and glycosylation takes place in the Golgi). In addition, no significant changes in nucleotide sugars were detected using an LC-MS approach, nor were changes in choline or choline phosphate levels detected. Further work is required to identify the natural substrate(s) of TcoDMT.

In summary, this study characterises and validates the first defined mechanism of resistance to the widely used trypanocide ISM in the disease-relevant species *T. congolense*, and thereby uncovers a novel eukaryotic phenanthridine transporter. TcoDMT is also the first genetic determinant definitively shown to regulate both ISM and EtB resistance in this parasite species. Furthermore, copy number of this transporter strongly correlates with ISM resistance in field isolates. A logical next step will be to develop and validate assays to monitor resistance in field isolates, likely via RT-qPCR to determine CNV. Should these experiments succeed, DMT will prove a useful marker in both regional and time-series studies to predict or monitor ISM sensitivity in *T. congolense* isolates and strains, with implications for disease management as well as informing stakeholders how to best maximise chemotherapeutic lifetime.

## Methods

### Parasite culture

*T. congolense* WT (wild-type), TcoDMT^OE^ (overexpression), TcoDMT^sKO^ (single knock-out) and TcoDMT^dKO^ (double knock-out) cell lines were grown at 34 °C, 5% CO_2_ and cultured in HMI-93^64^ supplemented with 20% goat serum (Gibco). Genetic manipulation was carried out as described below. TcoDMT^OE^ was selected and grown in 0.4 µg/mL blasticidin, TcoDMT^sKO^ in 0.4 µg/mL hygromycin, and TcoDMT^dKO^ in 0.4 µg/mL hygromycin and 0.4 µg/mL puromycin until knockout confirmation, after which the latter could be maintained in the absence of antibiotics. TcoDMT^RNAi^ cells were selected and grown in 0.4 µg/mL puromycin and 0.4 µg/mL G418 (neomycin), and RNAi was induced via the daily addition of 1 µg/mL tetracycline.

*T. b. brucei* Lister 427 WT and OE/dKO mutants, 2T1 and 2T1^T7-Cas9^ cell lines were cultured in HMI-11 medium^65^, and maintained at 37 °C and 5% CO_2_. TbDMT^OE^ was selected and grown in 5 µg/mL blasticidin, *T. brucei* 2T1 maintained in 0.5 µg/mL phleomycin and 0.2 µg/mL puromycin^66^ and the resulting TbDMT^RNAi^ selected with 0.5 µg/mL phleomycin and 2.5 µg/mL hygromycin after transfection^67^. *T. brucei* 2T1^T7-Cas9^ was grown in 2.5 µg/mL hygromycin and the generated *T. brucei* DMT-Cas9 cells selected and grown in 2 µg/mL phleomycin following transfection^44^. Tetracycline at 1 µg/mL was added to the culture daily in order to induce RNA interference and double strand break in TbDMT RNAi and TbDMT-Cas9 cell lines, respectively.

To determine parasite growth rate, cells were seeded at predetermined density and cell densities determined daily via haemocytometry. *T. congolense* were detached via pipetting prior to assessing densities.

### Parasite transfection

Both *T. congolense* and *T. brucei* were transfected with 10–15 µg linearised construct DNA. Briefly, per transfection, 2–4 × 10^7^ parasites were centrifuged at 1500 × *g* for 10 min. The medium supernatant was poured off, leaving ~1 mL. Cells were resuspended and transferred to 1.5 mL eppendorf tubes, and centrifuged for 5 min at 1500 × *g*. Remaining supernatant was removed, cells washed in phosphate-buffered saline (PBS; 137 mM NaCl, 3 mM KCl, 10 mM Na_2_HPO_4_, 1.8 mM KH_2_PO_4_), subsequently resuspended in 100 µL transfection buffer^68^ and transferred to an electroporation cuvette along with linearised vector, prior to electroporation using programme Z-001 on the Nucleofector II (Lonza). Cells were then transferred to 20 mL warm medium and allowed to grow. Selective antibiotics (as indicated above) were added after 24 h and cells were simultaneously cloned by diluting (1:50, 1:100 and 1:200) into 96-well plates. Resultant transfectants were isolated after ~7 days and were deemed clonal if <35 wells contained viable cells (based on a Poisson model of 1 cell per well).

### Parasite genetic manipulation

Unless stated otherwise, all cell line constructs were typically generated as follows: amplicons generated by PCR using *T. congolense* or *T. brucei* genomic DNA (gDNA) templates were first ligated into the pGEM-T easy vector using T4 DNA ligase (both Promega), prior to excision with construct-specific restriction enzymes (see Supplementary Data 4 for primer sequences and restriction enzyme information). Sanger sequencing was typically performed at this stage. Excised constructs were subsequently ligated into Antarctic phosphatase-treated (NEB) target vector using T4 ligase. Final vectors were linearised with plasmid-specific restriction enzymes prior to transfection into target cells as described above.

To generate the TcoDMT^OE^ line, a previously-generated vector targeting the *T. congolense* tubulin array for constitutive expression was used^34^ (Supplementary Data 4). The TcoDMT (gene ID: TcIL3000.A.H_000575200) ORF was amplified using primers containing ClaI and BamHI sites in the forward and reverse primer, respectively (Supplementary Data 4). The final vector was linearised using AscI prior to electroporation. To generate TcoDMT^dKO^ constructs, sequential allelic replacement was used, whereby CDS alleles are replaced with antibiotic selection cassettes via two rounds of transfections to generate a cell line resistant to two antibiotics. Replacement cassettes were generated by amplifying a region of DNA encompassing the 5’-untranslated region (UTR), using forward and reverse primers containing a NotI and an XbaI site, respectively. In addition, a region of DNA encompassing the 3’-UTR was amplified using forward and reverse primers containing an NsiI and an XhoI site, respectively (Supplementary Data 4). To ligate the constructs into the final vectors, two rounds of digestion and ligation were carried out, one for each UTR. Two plasmids were generated, each with a different antibiotic resistance cassette (hygromycin and puromycin) flanked by the aforementioned UTRs. Constructs were linearised with NotI and XhoI prior to transfection. The first transfection generated the TcoDMT^sKO^ line, selected using hygromycin. This line was then transfected with the second construct, containing a puromycin selection cassette, to generate the TcoDMT^dKO^ line. For the generation of the TcoDMT^RNAi^ construct, a previously-generated RNAi vector was used^33^. A 503 bp fragment of the TcoDMT gene was amplified from *T. congolense* IL3000 gDNA using forward and reverse primers flanked by FseI and HindIII sites, respectively (Supplementary Data 4). The final vector was linearised with NotI prior to electroporation.

In order to generate TbDMT^OE^ cells, the TbDMT gene (Tb927.8.1460) was amplified using forward and reverse primers containing XbaI and BamHI restriction sites respectively (Supplementary Data 4) and the cloned into the pGL2271^34^ final vector. The plasmid was linearised with AscI prior to electroporation. TbDMT RNAi stem-loop plasmids were constructed using two-step ligation with TbDMT fragment as described previously^67^. The forward primer contained KpnI and BamHI sites whilst the reverse primer containted ApaI and XbaI sites (Supplementary Data 4). After excision from pGEM-T easy, the gene fragments were cloned into the pRPa^iSL^ vector in two steps. In the first step, construct and vector were digested with KpnI and BamHI and ligated to generate an intermediate RNAi vector^67^. In the second step, the original construct as well as the aforementioned intermediate RNAi vector were digested using ApaI and XbaI and ligated together. Constructs were linearised with AscI and transfected into *T. brucei* 2T1 cells^66,67^. Inducible CRISPR/Cas9 dKO cell lines of TbDMT were generated using two separate sgRNA pairs as previously described^44^. Potential sgRNAs were identified using the LeishGEdit.net platform and manually selected based on PAM-adjacent GC-content. AGGG and AAAC overhangs were added to the 5’ end of the forward and reverse oligo sequences respectively. Synthesised oligos were annealed by heating to 95 °C followed by slowly cooling over 4 h, and annealed oligos were subsequently ligated into BbsI-digested pT7^sgRNA^ backbone. Constructs were linearised using NotI prior to electroporation into *T. brucei* 2T1^T7-Cas9^ cells^44^.

For N-terminal tagging of DMT protein with superfolder GFP by modification of the endogenous locus, PCR amplicons containing ~750 bp from the 5’-end UTR (untranslated region) and ~750 bp from the N-terminal end of the CDS (coding sequence) of DMT were amplified from *T. congolense* IL3000 gDNA and cloned together into XbaI-BamHI sites downstream of the fluorescent protein ORF in pEnNY0^69^ using standard methods (Supplementary Data 4). In the same step, a NotI linearization site was introduced between the UTR and CDS. For N-terminal tagging of ectopic TcoDMT expression (TcoDMT^OE-sfGFP^), the same vector used to generate TcoDMT^OE^ cells was further manipulated via insertion of sGFP at the 5’-end. The sfGFP ORF was amplified from the aforementioned pEnNY0 vector using primers flanked by ClaI sites and ligated into the TcoDMT^OE^ vector digested with the same enzyme. Of note, the resulting vector was transformed into methyltransferase deficient (*dam*^*–*^/*dcm*^*–*^; NEB) chemically competent *E. coli*, as ClaI is methylation-sensitive and the forward primer contained a potential CpG island. The resulting plasmid was linearised using AscI.

### Quantitative real-time PCR

Total RNA was extracted using a commercial kit (RNeasy; QIAgen) and a total of 1 µg RNA was reverse-transcribed using High-Capacity cDNA RT kit (Thermo Applied Biosystems). For RNAi studies, RNA samples were obtained from tetracycline-induced and uninduced samples daily for 3 days post-induction^7^. qPCR reactions were carried out with SensiFAST SYBR Hi-ROX mix (Bioline), in a BioRad Thermocycler (Biorad) as described^7^. Normalised transcript level was determined through the ΔΔCt method^70^ using TcoTERT and TbTERT as endogenous controls (Supplementary Data 4) for *T. congolense* and *T. brucei*, respectively^31,71^. To calculate TcoDMT copy number from genomic DNA, qPCR reactions were carried out as above using 2 ng gDNA. Expression levels, calculated using the ΔΔCt method, were then multiplied by 2 to account for the diploidy of the *T. congolense* genome.

### Alamar blue drug sensitivity assays

Drug sensitivities were determined using the resazurin-based Alamar blue assay as described previously^7,72^. Briefly, two-fold serial dilutions of the test drug including drug-free control were prepared in 100 μL of culture medium in 96-well white opaque flat-bottomed plates (Cell Star, Greiner Bio-one). Parasites at log-phase of growth were harvested and distributed across the wells containing the drug dilution series at 100 μL/well, with final concentrations of 2 × 10^4^ and 2.5 × 10^5^ cells/mL for *T. brucei* and *T. congolense*, respectively. Plates were incubated for 48 h at the relevant growth conditions for each species, after which 20 μL of 125 mg/mL (0.49 mM) resazurin dye in PBS was added to each well, and the plate further incubated for 24 h. The endpoint fluorescence at λ_em_544 nm/λ_exc_590 nm was determined for each well of the plate using a Cytation 5 imaging reader (BioTek), and the EC_50_ of each drug was calculated using non-linear regression fitted to a sigmoidal dose-response curve in GraphPad Prism (v10).

### Drug uptake assays

The rate of uptake of ISM was determined using the natural fluorescence of ISM as described^21^. Briefly, 1 × 10^7^ cells in assay buffer (33 mM HEPES, 98 mM NaCl, 4.6 mM KCl, 0.55 mM CaCl_2_, 0.07 mM MgSO_4_, 5.8 mM NaH_2_PO_4_, 0.3 mM MgCl_2_, 23 mM NaHCO_3_ and 14 mM glucose, pH 7.3) were incubated with 10 µM ISM for an intended duration, followed by centrifugation at 12,500 × *g* for 1 min to stop the reaction and to separate the cells from the drug mix through an oil layer (7:1 v/v dibutylphthalate/mineral oil; Sigma-Aldrich). The oil and drug mix were removed by capillary suction and 50 μL of a 0.1 N HCl/methanol (1:8 v/v) mixture added to the tube to lyse the cell pellet. Following 1 h incubation at room temperature, samples were transferred to a 96-well plate and the fluorescence determined at λ_em_620 nm/λ_exc_355 nm using a Cytation 5 imaging reader. Uptake was reported as percentage of the maximum fluorescence in arbitrary units recorded in WT samples^73^.

For the determination of radiolabelled ISM uptake, the cell pellet was collected and lysed as described previously for radiolabelled trypanocides^74^. Following incubation of cells with 1 µM [^14^C]-ISM (^14^C-Samorin), microcentrifuge tubes were flash frozen in liquid nitrogen, and the cell pellet cut off and transferred to a scintillation vial. Cell pellet was lysed in 2% SDS solution under slow agitation for 30 min and 3 mL of scintillation fluid (Scintilogic U, Lablogic) was subsequently added, followed by further incubation overnight in the dark. Radiation was measured in a 300SL scintillation counter (Hidex) and the rate of uptake per unit time was calculated using GraphPad Prism version (v10).

### Flow cytometry for the measurement of mitochondrial membrane potential

Mitochondrial membrane potential was assessed as described previously, with minor modifications^75^. Cells were harvested and adjusted to 1 × 10^6^ cells/mL in 1 mL HMI-93 medium, and samples were either treated with 500 nM ISM for 3 h, 200 nM valinomycin for 1 h, 20 µM troglitazone for 1 h or left untreated. Thereafter, all samples were treated with 0.05 µM Mitotracker Red (Thermo Fisher Scientific) and incubated for 15 min at 37 °C and 5% CO_2_. Cells were then washed in PBS at 1500 × *g* for 10 min, fixed in 4% paraformaldehyde for 5 min, then washed again and resuspended in PBS. Mitotracker Red fluorescence intensity was determined using a Fortessa X20 flow cytometer (BD Bioscience) set at λ_em_578 nm/λ_exc_599 nm and the data analysed using FlowJo software (BD Bioscience).

### Native fluorescence microscopy

For localisation of tagged DMT by native fluorescence, cells were harvested from mid-log phase cultures, washed twice in PBS and allowed to settle for 5 min onto glass slides at ~2 × 10^7^ cells/mL density^33^. Cells were fixed for 5 min in 2% (w/v) formaldehyde, permeabilised in −20 °C methanol, re-hydrated in PBS and incubated with 15 ng/mL 4,6-diamidino-2-phenylindole for 5 min, before mounting in 1% (w/v) 1,4-diazabicyclo[2.2.2]octane, 90% (v/v) glycerol, 50 mM sodium phosphate, pH 8.0^33^. Images were captured on an Olympus BX51 microscope equipped with a 100× UPlanApo objective (1.35 NA; Olympus) and Retiga R6 CCD camera (Qimaging) without binning. All fluorescent channel images were captured at equal exposure settings without prior illumination. Images for level comparison were also processed in parallel with the same alterations to minimum and maximum display levels. Image acquisition was controlled by μManager open source software^76^. Processing and analysis were performed in Fiji^77^. To plot pixel intensity profiles of native fluorescence images, a straight line covering 7 µm was drawn through the fluorescent panel. Data was imported into GraphPad Prism and the line was smoothed with the following parameters: Number of neighbours averaged: 5; Order of the smoothing polynomial: 2nd.

For ISM fluorescence, a final concentration of 10 µM ISM was added to cells in PBS at a density of 2 × 10^7^ cells/mL for 30 min prior to the aforementioned steps. Images were captured on a Zeiss Axio Observer Z.1 microscope equipped with a 63× Plan-Apochromat objective (1.4 NA; Zeiss) and a Axiocam 702 mono (Zeiss). All images were captured and processed as above.

### TcoDMT read depth in field isolate sequencing data

Genomic sequences of 56 *T. congolense* isolates were obtained from EBI under the accession number PRJEB15251, previously deposited by Tihon and colleagues^45^. Of these, 21 had associated metadata indicating sensitivity to ISM (Supplementary Data 3). These 21 genome sequences were subsequently aligned to the *T. congolense* IL3000 2019 reference genome sequence (v51; TriTrypDB) using Hisat2^78^ with the following parameters to ensure multimapping reads were aligned only once: “*-- no-spliced-alignment -k 1*”. Using Samtools^79^, the resulting alignment files were filtered (“*view -bS -q 1 -F 0×100*”) and sorted (“*sort*”) to generate .bam files.

To obtain normalised read depth for each of the 21 alignments, the “bamCoverage” function from the DeepTools suite^80^ was employed. The following parameters were used: the output format was bedgraph (*“--outFileFormat “bedgraph”*), a rolling bin size of 100 bp (“*--binSize 100*”) and RPKM normalisation (“*--normalizeUsing RPKM*”) and only chromosome 8 reads were normalised for analysis of the DMT region (*“--region Tc.A.H.pschr_08*”). For other loci, the relevant chromosome was selected (chromosomes 11, 9 and 10 for TcoTERT, TcoFBP and TcoALD, respectively). A custom R script was then used to generate rolling mean coverages (rmCoverage) for each bedgraph file. This script made use of the “rollapply” function from the “zoo” package^81^.

The “unionbedg” function from the bedtools suite^82^ was used to merge all RPKM normalised bedgraph into one file for downstream analyses. To generate figures, the “Gviz” package for R was used^83^. The “*ucscChromosomeNames* = *FALSE*” option was applied in order to utilise a custom *T. congolense* genome sequence using publicly available annotation and sequence files (v51; TriTrypDB). Figures were edited in Inkscape v1.2.

### In silico analysis of TcoDMT

The protein sequence of TcoDMT was analysed using InterProScan^84^ and domain graphics were generated using R. Colabfold^42^ was used to generate a structural predication of TcoDMT, with default parameters. The model with highest rank was uploaded in FoldSeek to search for similar 3D protein structures. Images of overlaid protein structures were downloaded from FoldSeek.

### Phylogenetics

Sequences for all *H. sapiens* SLC35 proteins were downloaded from Uniprot using the search term “SLC35*”. Previous studies identified eight NSTs in *T. brucei*^41^ (strain TREU 927) and 11 NSTs in *T. cruzi* (strain CL Brener Non-Esmeraldo-like). The protein sequences for these NSTs were used to identify NSTs in *T. congolense* and *L. major* (strain Friedlin) via BLASTp searches in TriTrypDB. To further confirm all NSTs had been identified, the predicted proteomes from the aforementioned parasite species were downloaded from TriTrypDB and compared using Orthofinder^85^. A multiple sequence alignment of NSTs and SLC35 sequences (total of 55) was generated using Clustal Omega Multiple Sequence Aligner^86^ with default settings and the Pearson/FASTA output format. Tree construction was carried out using iqtree2^87^ with 1000 bootstrap replicates (“*-B 1000*”). The resulting tree was annotated and rendered using iTOL^88^.

### Metabolite extractions and liquid chromatography-mass spectrometry

Metabolite extractions were carried out on both cell pellets and culture supernatants. Three replicates for each line were grown for 48 h to a final density of 2 × 10^6^ cells/mL, and 10^8^ cells were rapidly quenched to 4 °C in a dry ice/ethanol bath. All subsequent steps and incubations were carried out at 4 °C. Samples were centrifuged at 1250 × *g* for 10 min after which, for supernatant samples, 5 µL was taken from each sample and added to 200 µL extraction solvent (chloroform:methanol:water in a 1:3:1 ratio) in 1.5 mL eppendorf tubes. The remaining supernatant was discarded and cells resuspended in 1 mL sterile PBS. After transferring cell pellet samples to 1.5 mL eppendorf tubes, they were centrifuged at 1250 × *g* for 10 min. Supernatant was discarded and 200 µL extraction solvent (as above) was added to each sample. All samples were then incubated for 1 h on a thermomixer at 4 °C. Samples were centrifuged at 16,060 × *g* for 10 min, and supernatants (~195 µL) transferred to new eppendorf tubes. As controls, 10 µL from each metabolite extraction was added to a new tube. Finally, air in the tubes was displaced using argon gas, and samples stored at −80 °C prior to LC-MS analysis.

Hydrophobic interaction liquid chromatography (HILIC) was carried out on a Dionex UltiMate 3000 RSLC system (Thermo Fisher) using a ZIC-pHILIC column (150 mm × 4.6 mm, 5 µm column; Merck SeQuant), at the University of Glasgow Shared Research Facility, UK. The column was maintained at 25 °C and samples were eluted with a linear gradient (20 mM ammonium carbonate in water and acetonitrile) over 26 min at a flow rate of 0.3 mL/min. Injection volume was 10 µL and samples were maintained at 5 °C prior to injection. For MS analysis, a Thermo Orbitrap QExactive (Thermo Fisher Scientific) was operated in polarity switching mode. The MS settings were as follows: Resolution: 70,000; AGC: 1e6; m/z range: 70–1050; Sheath gas: 40; Auxiliary gas: 5; Sweep gas: 1; Probe temperature: 150 °C; Capillary temperature: 320 °C. For positive mode ionisation: source voltage +3.8 kV, S-Lens RF Level 30.00, S-Lens Voltage 25.00 (V), Skimmer Voltage 15.00 (V), Inject Flatopole Offset 8.00 (V), Bent Flatapole DC 6.00 (V). For negative mode ionisation: source voltage-3.8 kV. A set of authentic standards was run prior to the sample set.

### Metabolomics analysis

Metabolomics data obtained in raw format were converted first to mzXML format and split into positive and negative polarity using msconvert^89^. Files were then converted to peakML format using XCMS, and were further processed using mzMatch^90^. Peak annotation and metabolite identification was carried out using IDEOM^91^. Data were analysed using MetaboAnalyst (v6.0)^92^. Prior to analysis, data were transformed (Log_10_) and pareto-scaled.

### Computational methods

Statistical analyses were carried out both in GraphPad Prism and R^93^, depending on the experimental procedure. For cartoon graphics (Fig. 5), icons were downloaded from Bioicons. These icons were created by Servier (https://smart.servier.com) and are licensed under CC-BY 3.0 Unported (https://creativecommons.org/licenses/by/3.0).

## Supplementary information

Supplementary Information

Supplementary Data 1

Supplementary Data 2

Supplementary Data 3

Supplementary Data 4

## Data Availability

All data generated or analysed during this study are included in this published article and its Supplementary Information files.
