# Supplementary material for: A cell surface transporter mediates phenanthridine resistance in African trypanosomes
Source: NPJ Antimicrob Resist. 2026 Jul 4;4:62. doi: 10.1038/s44259-026-00228-1 (PMC13642254; doi:10.1038/s44259-026-00228-1)
Supplement: Supplementary file 1 [file 44259_2026_228_MOESM1_ESM.pdf]

Supplementary data: Steketee et al., *A cell surface transporter mediates phenanthridine resistance in African trypanosomes*

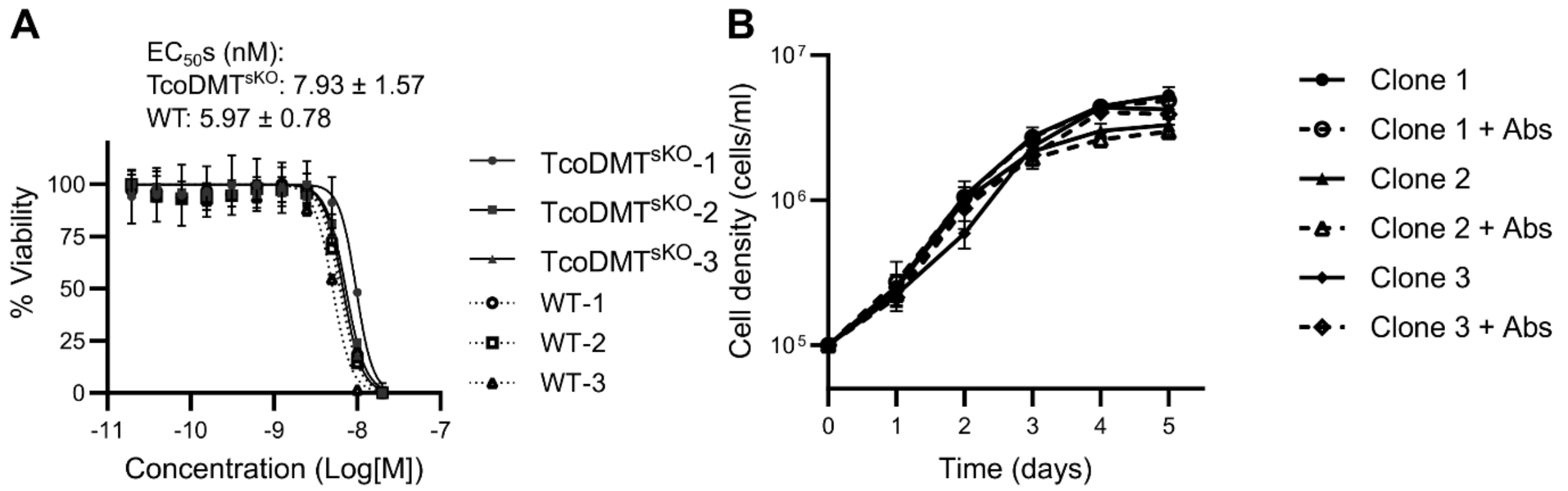

Figure S1: Generation of an intermediate TcoDMT hemizygous knock-out (TcoDMT^{sKO}) A) Sigmoidal dose-response curves for ISM treatment of *T. congolense* IL3000 WT and TcoDMT^{sKO} cells to determine the effect of single allelic knock-out of TcoDMT on ISM sensitivity. B) Growth of TcoDMT^{dKO} cells in the presence and absence of selective antibiotics (Abs). Parasites were maintained in the absence or presence of 0.4 µg/mL hygromycin and 0.4 µg/mL puromycin. No differences were observed between the two sample groups.

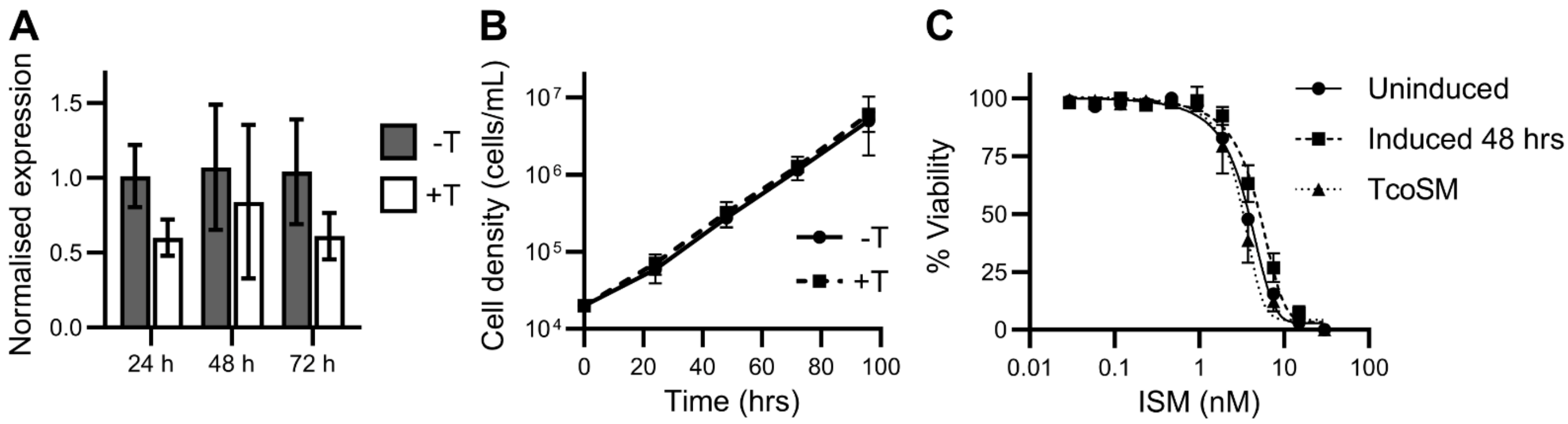

Figure S2: Effect of TcoDMT RNAi-mediated knock-down on ISM sensitivity. A) RT-qPCR analysis of tetracycline-induced (+T) and uninduced (-T) TcoDMT RNAi knock-down, highlighting a loss of TcoDMT expression of ~40%. B) Growth curves of tetracycline-induced (+T) and uninduced (-T) TcoDMT RNAi cells. C) Sigmoidal dose-response curves of TcoDMT RNAi cells, in the absence or presence of tetracycline (1 µg/mL), in addition to the TcoSM background strain. Cells induced with tetracycline were assayed after 48 hours of induction, highlighting that RNAi of TcoDMT had no effect on ISM sensitivity.

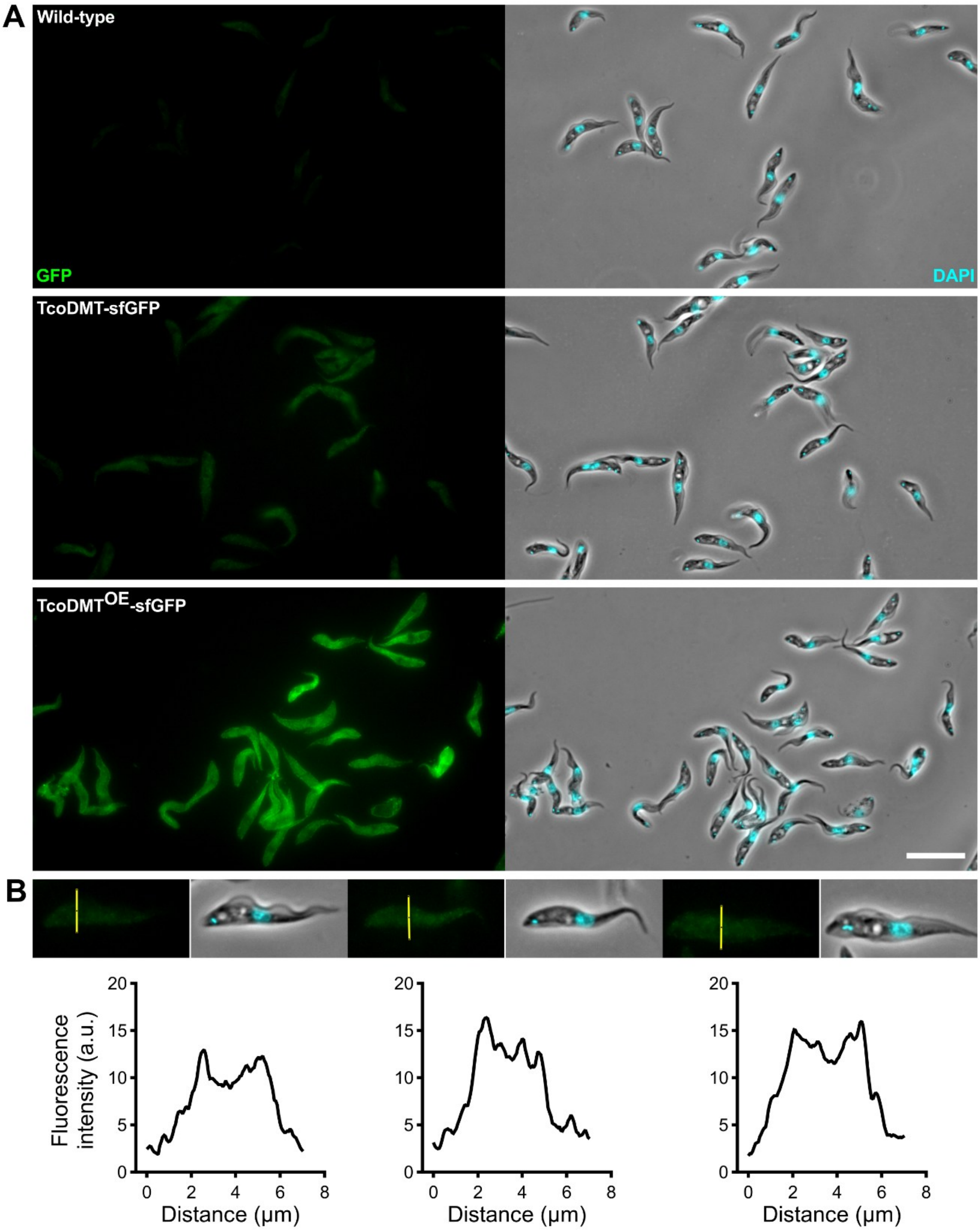

Figure S3: Native fluorescence analysis of TcoDMT localisation. A) Representative images of wild-type (top), TcoDMT^{sfGFP} (middle) and TcoDMT$^{OE\text{-}sfGFP}$ (bottom) cells. GFP (green) fluorescence, specific for TcoDMT, is shown on the left, and phase-contrast merged with DNA staining (cyan) is shown on the right. Scale bar represents 20 µm. B) Pixel intensity analysis of TcoDMT^{sfGFP}. The three examples are also shown in Fig 2. Line profiles (indicated by yellow lines) were generated to determine relative protein abundance across the cell. Resulting plots (underneath each micrograph) highlight intensity patterns that are typical of cell surface proteins.

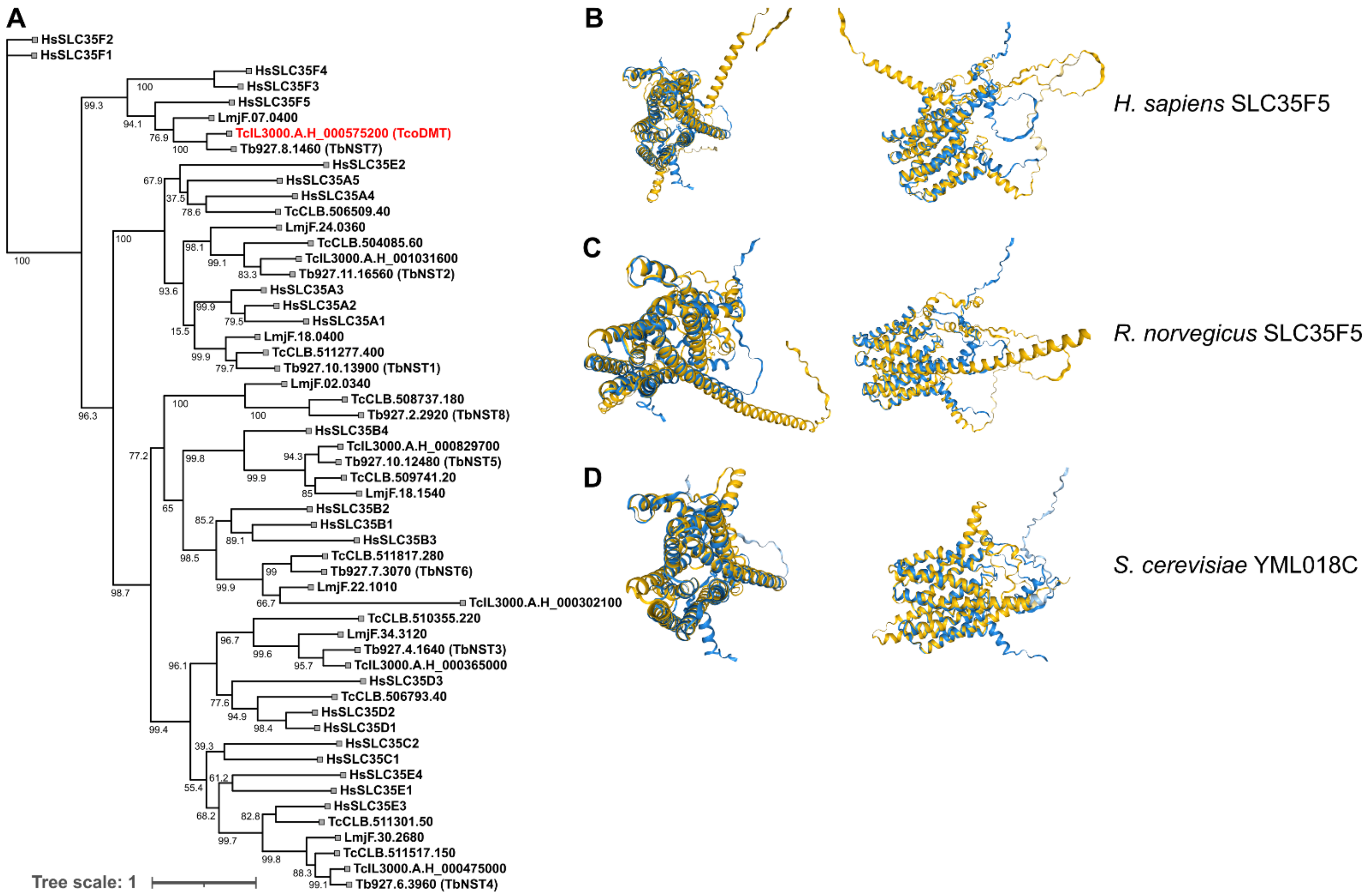

Figure S4: Analysis of TcoDMT phylogeny and structure. A) Phylogenetic tree of the *H. sapiens* SLC35 family and known sugar nucleotide transporters (nucleotide sugar transporters; NSTs) from *T. brucei*, *T. cruzi* and *Leishmania major*, to highlight closest relatives of the TcoDMT protein. B-D) FoldSeek analysis of the predicted TcoDMT structure. TcoDMT is structurally similar to predicted structures of SLC35F5 proteins in *H. sapiens* (B) and *R. norvegicus* (C), as well as the *S. cerevisiae* uncharacterised vacuolar membrane protein YML018C (D). Two different views are shown for each structural alignment and in each example, TcoDMT is coloured blue, with the compared protein structure in yellow. Protein structure similarity metrics of TcoDMT compared to each protein: *H. sapiens* SLC35F5: TM-score: 0.57697, RMSD: 20.29; *R. norvegicus* SLC35F5: TM-Score: 0.58773, RMSD: 19.61; *S. cerevisiae* YML018C: TM-Score: 0.73935, RMSD: 7.36.

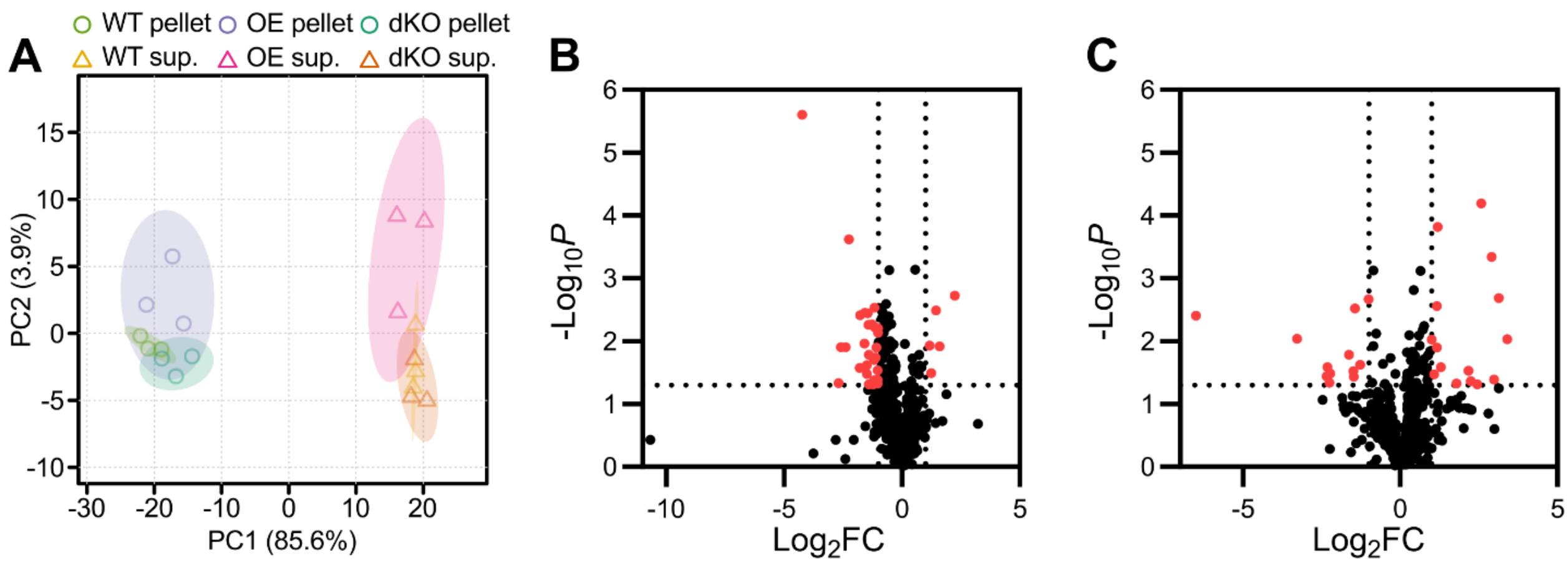

Figure S5: Metabolomics analysis of TcoDMT mutants. A) PCA plot of processed LC-MS metabolomics samples (WT, TcoDMT^{OE}, TcoDMT^{dKO}) highlighting separation between cell pellet samples ("pellet") and supernatant samples ("sup.") after 48 hours of *in vitro* culture. Given the lack of separation between WT and TcoDMT^{dKO} cells, the WT vs TcoDMT^{OE} comparison was analysed in further detail. B) Volcano plot showing fold changes (TcoDMT^{OE} / WT) in metabolites detected in cell pellet samples. Of the 950 metabolites, 28 were significantly reduced and 5 were significantly increased in abundance, as indicated by red dots (significance calculated by Student's *t*-test). C) Volcano plot showing fold changes (TcoDMT^{OE} / WT) in metabolites detected in supernatants after 48 hours of cell culture. Of the 774 metabolites detected, 15 were significantly increased in abundance, suggesting these metabolites were excreted more rapidly or consumed to a lesser degree by TcoDMT^{OE} cells. Conversely, 12 metabolites were significantly reduced in TcoDMT^{OE} supernatants compared to WT supernatants, indicating that these metabolites were either consumed more rapidly, or excreted less rapidly.

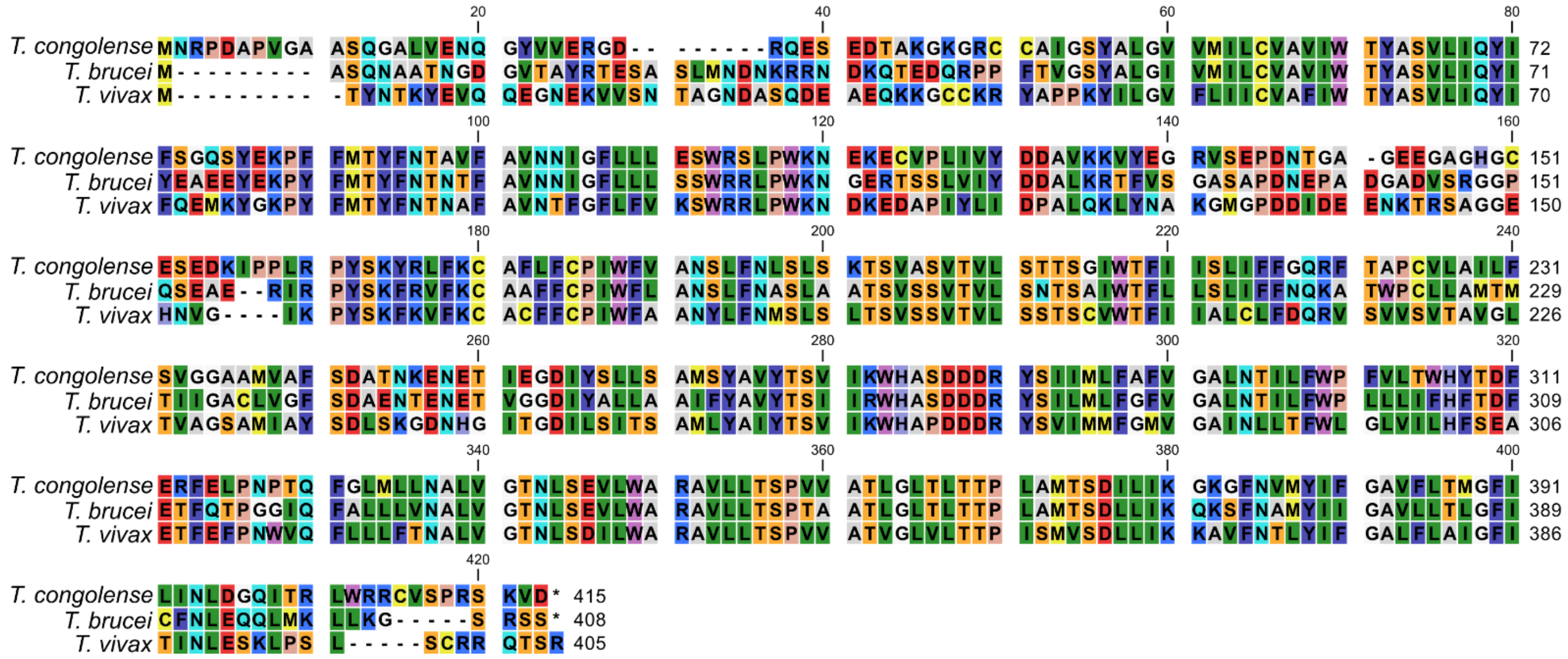

Figure S6: Protein sequence alignment of DMT. Sequences are shown for TcoDMT (TcIL3000.A.H_000575200), TbDMT (Tb927.8.1460) and TvDMT (TvY486_0800880) using Rasmol colouring.

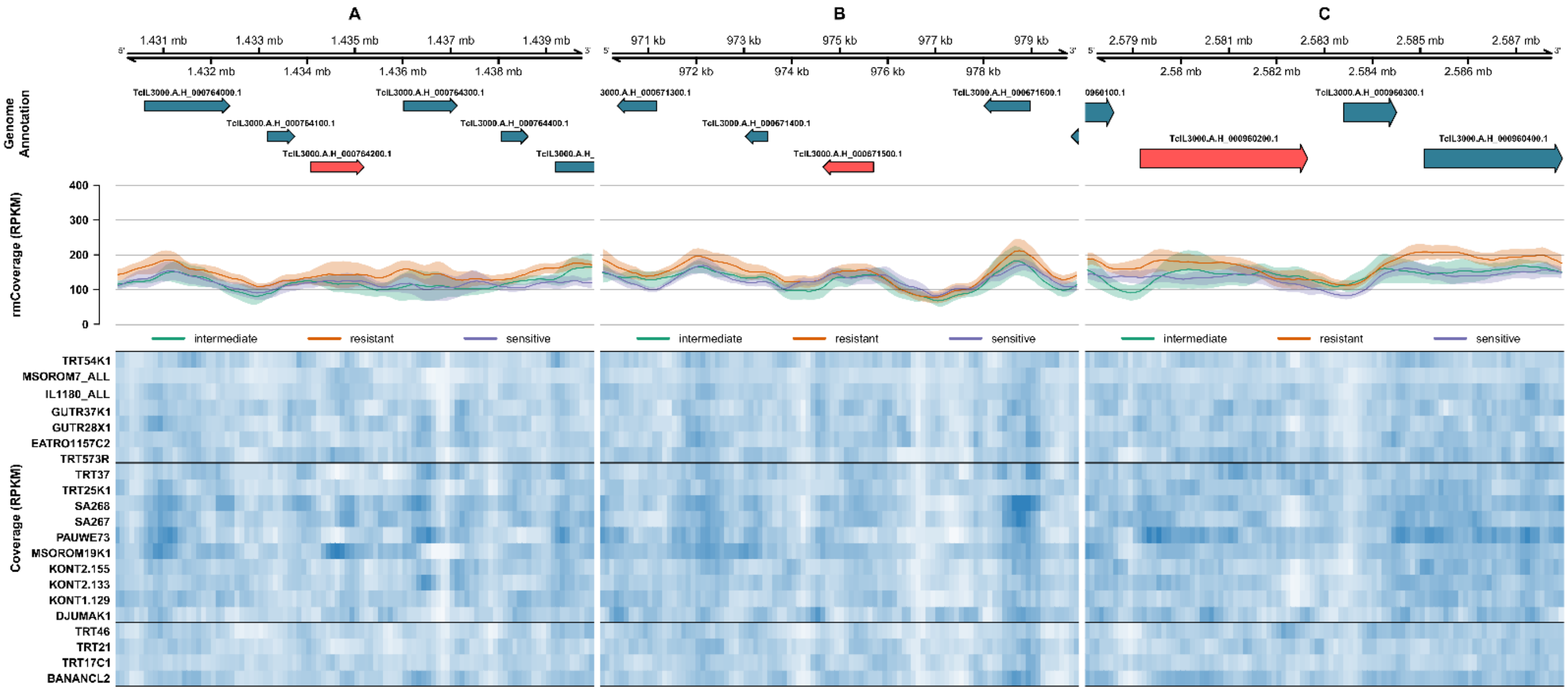

Figure S7: Read coverage of sensitive, intermediate and fully resistant field isolates in three unrelated loci encompassing single copy genes. Genes are highlighted in red in the Genome Annotation panel. A) Aldolase (ALD; TcIL3000.A.H_000764200.1); B) Fructose-1,6-bisphosphatase (FBPase; TcIL3000.A.H_000671500.1); C) Telomerase reverse transcriptase (TERT; TcIL3000.A.H_000960200.1).

Supplementary Data 1: FoldSeek output assessing similarity of the TcoDMT predicted structure to other predicted and solved structures. Results are partitioned into worksheets for each database source (AFDB, Alphafold Database; PDB100, Protein Data Bank; MGNIFY/GMGCL, Metagenomic protein structural clusters). Abbreviations: Prob: probability; Seq Id: sequence identity; E-value: expect value; Query/Target Pos: Query/Target position.

Supplementary Data 2: LC-MS metabolomics analysis of *T. congolense* WT, $TcoDMT^{OE}$ and $TcoDMT^{dKO}$ lines, including both cell pellets and supernatants after 48 hr of *in vitro* culture. Metabolites identified via authentic standards included in the same experiment are highlighted in yellow (MS level 1 identification). Columns L-Q show fold changes between samples groups, columns R-U show Log_2 fold changes between DMT mutants and WT sample groups. Raw intensity values are shown in columns V-AM. Abbreviations: WT: wild-type; TcoDMT-OE: TcoDMT overexpressor; TcoDMT_dKO: TcoDMT double knock-out; cp: cell pellet; sn: supernatant

Supplementary Data 3: Metadata and references for field isolate samples used in TcoDMT copy number analysis. Run accession (column A) refers to the accession as listed on the European Nucleotide Archive. References describing the original field isolate can be accessed via hyperlinks in column C. ISM sensitivity of each field isolate is indicated in column D (red for resistant, blue for sensitive).

Supplementary Data 4: List of primers used in this study.
